# Comparative Genomic Analysis of *Sulfurospirillum cavolei* MES Reconstructed from the Metagenome of an Electrosynthetic Microbiome

**DOI:** 10.1371/journal.pone.0151214

**Published:** 2016-03-16

**Authors:** Daniel E. Ross, Christopher W. Marshall, Harold D. May, R. Sean Norman

**Affiliations:** 1 Department of Environmental Health Sciences, Arnold School of Public Health, University of South Carolina, Columbia, South Carolina, United States of America; 2 Department of Microbiology & Immunology, Marine Biomedicine & Environmental Science Center, Medical University of South Carolina, Charleston, South Carolina, United States of America; Illinois Institute of Technology, UNITED STATES

## Abstract

*Sulfurospirillum* spp. play an important role in sulfur and nitrogen cycling, and contain metabolic versatility that enables reduction of a wide range of electron acceptors, including thiosulfate, tetrathionate, polysulfide, nitrate, and nitrite. Here we describe the assembly of a *Sulfurospirillum* genome obtained from the metagenome of an electrosynthetic microbiome. The ubiquity and persistence of this organism in microbial electrosynthesis systems suggest it plays an important role in reactor stability and performance. Understanding why this organism is present and elucidating its genetic repertoire provide a genomic and ecological foundation for future studies where *Sulfurospirillum* are found, especially in electrode-associated communities. Metabolic comparisons and in-depth analysis of unique genes revealed potential ecological niche-specific capabilities within the *Sulfurospirillum* genus. The functional similarities common to all genomes, *i*.*e*., core genome, and unique gene clusters found only in a single genome were identified. Based upon 16S rRNA gene phylogenetic analysis and average nucleotide identity, the *Sulfurospirillum* draft genome was found to be most closely related to *Sulfurospirillum cavolei*. Characterization of the draft genome described herein provides pathway-specific details of the metabolic significance of the newly described *Sulfurospirillum cavolei* MES and, importantly, yields insight to the ecology of the genus as a whole. Comparison of eleven sequenced *Sulfurospirillum* genomes revealed a total of 6246 gene clusters in the pan-genome. Of the total gene clusters, 18.5% were shared among all eleven genomes and 50% were unique to a single genome. While most *Sulfurospirillum* spp. reduce nitrate to ammonium, five of the eleven *Sulfurospirillum* strains encode for a nitrous oxide reductase (*nos*) cluster with an atypical nitrous-oxide reductase, suggesting a utility for this genus in reduction of the nitrous oxide, and as a potential sink for this potent greenhouse gas.

## Introduction

The genus *Sulfurospirillum* consists of heterotrophic denitrifiers and several species are capable of dissimilatory selenate and arsenate reduction [1], and/or reductive dehalogenation [1–5]. Members of the *Sulfurospirillum* genus have been found in many diverse ecosystems, including aquifer sediments [6], oil fields [7,8], and groundwater contaminated with chlorinated solvents [5], and in general are poorly characterized. *Sulfurospirillum spp*. are metabolically versatile and are assumed to play an important role in the cycling of sulfur and nitrogen, which is ascribed to many free-living *Epsilonproteobacteria* [9]. This activity has a direct effect on the cycling of iron, with various sulfur species acting as redox partners/electron shuttles between sulfur and iron. In a recent example, *S*. *deleyianum* was unable to reduce iron directly but utilized elemental sulfur, thiosulfate or sulfite as terminal electron acceptors, resulting in abiotic iron reduction via reduced sulfur species [10].

In this study, we have reconstructed a *Sulfurospirillum* draft genome [11] from the metagenome of an electrosynthetic microbial community [12,13], and performed genome comparisons in relation to other sequenced *Sulfurospirillum*. *Sulfurospirillum* was targeted for genome analysis due to its overall abundance within the microbial electrosynthesis system (MES) and potential role in long-term functional reactor operation [12,13]. Earlier studies by Marshall and coworkers examined the relative abundance of microbial taxa based upon conserved marker genes (bacterial 16S rRNA), which revealed that *Sulfurospirillum* comprised >90% of the active supernatant community and ~30% of the active cathode community [13]. This study, using shotgun sequencing of the metagenome and reconstruction of the full-length 16S rRNA gene, revealed that *Sulfurospirillum* represented ~20% of the electrode-associated microbial community and ~60% of the supernatant microbial community. *Sulfurospirillum* persisted in the MES at relatively high levels throughout the duration of the experiment, which suggests it plays an important role in the electrosynthetic community.

Since the first published *Sulfurospirillum* genome [14], a number of complete and draft *Sulfurospirillum* genomes have been published (Table 1) [11,15–17]. The total number of publicly available sequenced *Sulfurospirillum* genomes at the time of this study was eleven (5 complete, 6 draft). Recent work by Goris and coworkers has provided a thorough examination of *Sulfurospirillum multivorans* and a framework for comparative genomics within the *Sulfurospirillum* genus [17]. In this study we have expanded upon the work by Goris and co-workers with inclusion of eleven publicly available genomes to 1) obtain a more thorough characterization of *S*. sp. MES (Accession number: JSEC00000000) [11], to understand its role in electrosynthesis systems [12,13], and 2) increase the knowledge of the metabolic capabilities of the genus, highlighting both conserved and divergent metabolisms found in the *Sulfurospirillum* genus. Based upon updated phylogenomic analysis described herein, which now includes two recently sequenced *S*. *cavolei* strains, *S*. sp. MES should be designated as *S*. *cavolei* MES. Pan-genome analysis of eleven *Sulfurospirillum* strains revealed the presence of 6246 total gene clusters, 3191 unique gene clusters and 1082 gene clusters common across all eleven strains (core genome), shedding light on the overall shared genetic potential of this genus. Analysis of the functional metabolic pathways suggests many commonalities in general functions as well as unique gene sets that may have evolved due to ecological pressures. Interestingly, five of the eleven genomes examined encode for a nitrous-oxide reductase with accessory *nos* proteins, suggesting inclusion of *Sulfurospirillum* as a non-denitrifying atypical nitrous oxide reducer [18].

**Table 1:** Eco-physiological characteristics of members from genus *Sulfurospirillum*.

Species	Originated from	Metabolism	Reference
Sulfurospirillum deleyianum* **(CP001816.1)**	Anoxic mud from a forest pond	Sulfur reducing, dissimilatory nitrate reduction	[14,19–21]
***Sulfurospirillum multivorans* (CP007201.1)**	Activated sludge	able to reduce tetrachloroethene to *cis*-dichloroethene	[2,17,21,22]
***Sulfurospirillum arcachonense* (JFBL00000000) (draft)**	Oxidized marine surface sediment	microaerophilic sulfur-reducing	[23,24]
***Sulfurospirillum barnesii* (CP003333.1)**	Selenate-contaminated freshwater marsh	able to reduce selenate to elemental selenium	[1,20,21]
***Sulfurospirillum arsenophilum* NBRC 109478 (draft) (PRJDB1374)**	Arsenic-contaminated freshwater sediments	able to reduce arsenate to arsenite	[20,21]
*Sulfurospirillum halorespirans*	Anaerobic soil polluted with chlorinated aliphatic compounds	able to reduce tetrachloroethene to *cis*-dichloroethene	[1]
*Sulfurospirillum cavolei* strain Phe91	Underground crude oil storage cavity	facultatively anaerobic sulfur-reducing	[25]
***Sulfurospirillum cavolei* NBRC 109482 (draft) (BBQE00000000.1)**	Petroleum contaminated groundwater	—	—
***Sulfurospirillum* sp. SCADC (draft) (JQGK00000000.1)**	Methanogenic alkane degrading enrichment culture from an oil sands tailings	—	[16]
***Sulfurospirillum* strain Am-N (draft) (IMG object ID 2502171155)**	*Alvinella pompejana*, 13°N East Pacific Rise	—	[26]
***Sulfurospirillum* sp. strain MES (*S*. *cavolei* MES) (draft) (JSEC00000000)**	Metagenome of a microbial community enriched in an acetogenic microbial electrosynthesis system	—	[11–13]
***Sulfurospirillum* sp. strain UCH001 (AP014723.1)**	Chloroethenes-contaminated groundwater in Japan.	—	[15]
***Sulfurospirillum cavolei* UCH003 (AP014724.1)**	Chloroethenes-contaminated groundwater in Japan.	—	[15]

*S. *deleyianum* is the type species of the genus *Sulfurospirillum*. **Bolded species have a complete or draft genome sequence available**

## Materials and Methods

### Biological Sample

The initial reactor inoculum was obtained from a brewery wastewater basin (Charleston, SC). Supernatant and granule samples were extracted from a MES actively synthesizing acetate and hydrogen for over 150 days, under a constant stream of 100% CO_2_ and a cathode potential of -590 mV versus SHE [12,13].

### Sample processing

Nucleic acid (DNA/RNA) was processed in a similar manner to protocols of Poretsky and coworkers [27] and Gifford and coworkers [28]. Samples for nucleic acid extraction (culture supernatant or graphite granules) were aseptically and anaerobically removed from MESs. Culture supernatant (40 mL) was filtered through a 0.22 μm Sterivex^TM^ GP filter unit (Millipore) and graphite granules (~20 g) were placed into a 50 mL conical tube. All samples were subsequently frozen in liquid nitrogen and placed at -80°C until further processing. Supernatant filters and granule samples were removed from -80°C and placed on ice. RLT Plus buffer (Qiagen) was added to frozen granules or supernatant filters at a ratio of 1 mL RLT Plus to 4 mL granules or to one supernatant filter. β-mercaptoethanol (10 μL per mL of RLT Plus), and silicon carbide beads (DNase- and RNase-free mixture of 0.1 mm and 1 mm beads) were added to the RLT Plus buffer/sample mixture. Samples were incubated at room temperature for 10 min followed by 10 minutes of vigorous shaking (bead-beating) using a Mo-Bio Vortex Genie 2 with appropriate adapter. Samples were then subjected to five freeze/thaw cycles consisting of freezing in liquid nitrogen and thawing at 55°C. Following the freeze/thaw cycles, samples were transferred to a 15 mL conical tube and centrifuged at 9,300 rcf for 10 min at 4°C.

### DNA extraction

DNA was further purified from freeze-thaw lysed samples using an Allprep DNA/RNA Mini Kit (Qiagen). Extracted DNA was ethanol precipitated and resuspended in TE buffer (or ddH_2_O). DNA was quantified with a Nanodrop Spectrophotometer (Thermo Scientific, Wilmington, DE, USA), and a Qubit fluorometer (Invitrogen). The quantity of DNA ranged from 3.6 μg to 29.8 μg for supernatant and granule samples, respectively, and was used directly for downstream metagenome sequencing.

### Metagenome sequencing and processing

Purified genomic DNA, isolated from MES supernatant or MES cathode granules, was sequenced using the Illumina MiSeq platform and the Pacific Biosciences RS platform to obtain short and long reads, respectively. Read statistics can be found in Table A in S1 File.

### Illumina MiSeq platform

Extracted and purified DNA was sheared using Covaris adaptive focused acoustic technology and Illumina sequencing libraries were prepared using TruSeq LT DNA Sample Preparation Kits. The resultant library had an average size of 813 bp (supernatant) or 598 bp (electrode). Samples were sequenced using the Illumina MiSeq paired-end platform (2 x 250 bp). Two separate sequencing runs were performed and in total, over 30 million reads were generated with approximately 91% of the total bases sequenced with a quality score ≥ 30.

### Pacific Biosciences (PacBio) platform

Samples were prepared for PacBio sequencing according to manufacturer’s instructions (Pacific Biosciences) and protocols from the Interdisciplinary Center for Biotechnology Research (ICBR) at the University of Florida. Samples were subsequently sequenced on the PacBio RSII platform (P4-C2 chemistry) using three SMRT cells per sample with 2x55 minute movie times according to manufacturer’s protocol (Pacific Biosciences). The cathode (10 kb library) and supernatant (2 kb library) samples generated a total of 458,866 and 407,743 reads, respectively. The SMRTbell library was purified from hairpin dimers by two consecutive AMPure purifications using pre-washed Agencourt AMPureXP beads (Beckman Coulter). Read filtering, and metagenome assembly and analysis was performed on the total reads generated from all three SMRT cells for each sample.

### Metagenome/genome assembly

The metagenome/genome assembly has been briefly described in a genome announcement [11]—below is an in depth description of the methods used. Approximately 31-million metagenome sequence reads (7.2 billion total bases, 48.9% G+C content) were generated using the Illumina MiSeq platform with 2 x 250 bp paired-end sequencing and have been deposited in MG-RAST as 4673464.3–4673467.3 (ARPA_metagenome) (http://metagenomics.anl.gov/linkin.cgi?project=15936) and in the NCBI SRA database under Bioproject PRJNA245339. Longer sequencing reads (~1600 bp) were generated using a PacBio RS sequencer (Pacific Biosciences), with an output of 866,609 total reads (cathode and supernatant samples combined) and ~1.4 billion total bases. PacBio raw reads were deposited in the NCBI SRA database under Bioproject PRJNA245339. Illumina reads were trimmed based on quality (at both the 5’ and 3’ end) using CLC genomics workbench (CLC Genomics). PacBio reads were error corrected with the trimmed Illumina reads using PacBioToCA [29]. Trimmed Illumina reads were assembled in Velvet with a k-mer size of 61 [30]. The contigs and unmapped reads from the Velvet assembly were re-assembled with the trimmed Illumina reads and the error-corrected PacBio reads using the CLC genomics workbench (default settings). The metagenome assembly yielded an N50 of 67,989 with the max contig length of 724,237 using a total of 49,633,804 bases and a GC content of 56.6.

Preliminary binning of the genomes was performed using kmer coverage and GC content. The metagenome bins were refined using tetranucleotide frequencies [31]. PacBio reads were binned based on BLASTn results with default settings [32]. The *Sulfurospirillum-*associated reads were pooled and re-assembled using SPAdes Genome Assembler (default settings, k-mer sizes of 21, 33, 55, 77, 99, and 127; [33]). Quality of the draft genome in terms of completeness and contamination was assessed with Quast and CheckM [34,35]. Lineage-specific co-located marker sets were used to estimate genome completeness and contamination. Specifically, 115 reference genomes were utilized to infer marker sets [35]. A total of 397 inferred marker genes (390 inferred marker genes were identified once in the genome and 7 marker genes were identified twice) (Table L in S1 File) and 261 inferred co-located marker sets were utilized to estimate genome completeness at 100% and contamination at 2.38%. Contigs from the metagenome assembly most closely related to *Sulfurospirillum* were used for downstream genome assembly. Illumina reads and corrected Pacific Biosciences (PacBio) reads (Table A in S1 File) were mapped to the *Sulfurospirillum* contigs. The mapped reads were combined with the *Sulfurospirillum* contigs and re-assembled with SPAdes [33]. The input for the final hybrid genome assembly contained *Sulfurospirillum* contigs, *Sulfurospirillum* trimmed PacBio reads, and *Sulfurospirillum* Illumina reads. The final genome assembly generated 130 total contigs (of which 61 ≥ 500 bp and 48 ≥ 1000 bp) with an N50 of 371,847 bp; the longest contig was 724,139 bp (Table B in S1 File).

The genome assembly was assessed for quality based upon the cumulative length of the scaffolds and fold genome coverage (Fig A in S1 File). The majority of the genome (>94%) was contained within the 20 longest contigs (Fig A in S1 File). Using lineage-specific marker sets in the CheckM software [35] to determine genome completeness and contamination, the current assembly was characterized as being of ‘exceptional quality’ in terms of completeness (100%) and had ‘low’ contamination (2.38%), based upon the ‘controlled vocabulary’ used to describe the quality of a draft genome put forth by Parks and coworkers [35]. Marker sets (397) used to assess completeness and contamination can be found in Table L in S1 File. The draft genome consisted of 2.67 Mbp with a mean GC content of 43.8%. The genome size is similar to other sequenced *Sulfurospirillum* (*S*. *cavolei* UCH003 = 2.69 Mbp) and the GC content is similar to *S*. *cavolei* strain Phe91 (42.7%), *S*. *cavolei* UCH003 (43.9%) [15] and identical to *S*. *cavolei* NBRC (GCA_00813325.1) (43.8%) (Table C in S1 File). The assembly had 2,656 predicted unique genes based on quality assessment using QUAST [34] and is comparable to other *Sulfurospirillum* based on the number of predicted genes (Table C in S1 File). Gene annotation with the online Rapid Annotation using Subsystems Technology (RAST) [36] and other gene calling programs yielded similar results (Table B in S1 File). Initial annotation of the *Sulfurospirillum cavolei* MES genome was performed with RAST [36]. Further manual curation of specific pathways encoded by the *S*. *cavolei* MES genome was completed by cross-referencing with pathways from *S*. *multivorans* [17]. Specifically, a gene was identified from the *S*. *multivorans* genome and searched against the *S*. *cavolei* MES genome. Once the protein encoded by the gene of interest was identified, a PSI-BLAST (RAST default settings) was performed to determine the top three hits from the NCBI database (Table J in S1 File).

### Phylogenomic Analysis

Near full-length 16S rRNA gene sequences were identified using EMIRGE [37] with read mapping to the SILVA database and read annotation with BLASTn against the SILVA small subunit ribosomal RNA non-redundant database (http://www.arb-silva.de/) [37,38]. The phylogeny of the uncultured *Sulfurospirillum cavolei* MES was determined using the EMIRGE-generated near full-length 16S rRNA gene [37]. Full-length 16S rRNA gene sequences of other closely related cultured and uncultured *Sulfurospirillum* were downloaded from RDP and NCBI databases. Sequences were aligned with ClustalW and the ends were trimmed to generate sequences of uniform length (1390 bp with gaps). Trimmed sequences were re-aligned and an unrooted phylogenetic tree of the partial 16S rRNA gene was constructed using MEGA with the Jukes-Cantor distance model and neighbor-joining algorithms with a bootstrap value of 1000 [39].

A common technique to determine the degree of similarity between two organisms is to determine the extent of DNA hybridization of their genomes [40]. We identified the similarity of *Sulfurospirillum cavolei* MES to other *Sulfurospirillum* genomes through culture-independent *in silico* methods. The eleven genomes of the sequenced *Sulfurospirillum* (5 complete, 5 draft, and *Sulfurospirillum cavolei* MES) were compared structurally (Genome-to-genome Distance Calculator; CONTIGuator; RAST; ANI) and functionally (RAST; AAI) using a variety of open-source bioinformatics tools (Table D in S1 File) [36,41,42]. The complete genome sequences for *S*. *barnesii* (CP003333.1) [20], *S*. *deleyianum* (CP001816.1) [14], *S*. *multivorans* (CP007201.1) [2,17], *S*. *cavolei* UCH003 (AP014724) [15], and *S*. sp. UCH001 (AP014723) [15], and the draft genome sequences of *S*. *arcachonense* (JFBL00000000) [23], *S*. sp. SCADC (JQGK00000000.1) [16], *S*. *arsenophilum* NBRC (PRJDB1374), *S*. *cavolei* NBRC (BBQE00000000.1) and *S*. *cavolei* MES (JSEC00000000-formerly *S*. sp. MES) [11] were obtained from the National Center for Biotechnology Information (NCBI) GenBank database [43]. The draft genome of *S*. strain Am-N (IMG object ID 2502171155) was obtained from the Joint Genome Institute (JGI). Each draft genome was analyzed for completeness and contamination (as described above for *Sulfurospirillum cavolei* MES) using CheckM [35]. The *S*. Am-N draft genome was estimated to be 98.04% complete with 2.27% contamination. The *S*. *arcachonense* draft genome was estimated to be 99.62% complete with 0.00% contamination. The *S*. SCADC draft genome was estimated to be 100% complete with 0.97% contamination. The *S*. *cavolei* NBRC draft genome was estimated to be 100% complete with 4.29% contamination. The *S*. *arsenophilum* NBRC draft genome was estimated to be 99.62% complete with 0.65% contamination.

Digital DNA-DNA hybridization (DDH) was used to calculate genome-to-genome distances [44]. To gain structural insights (i.e. gene synteny) the *S*. *cavolei* MES draft genome assembly was mapped to the complete genome of *S*. *cavolei* UCH003, using the CONTIGuator software tool with default settings ([41]; Blast e-value = 1e^-20^; contig length threshold = 1000; contig coverage threshold % = 20; hit length threshold = 1100; multiple replicon threshold = 1.5; gap size for overlapping contigs = 100). The average nucleotide identity (ANI) was calculated using both best hits and reciprocal best hits between two genomes ([42]; default parameters—minimum length = 700 bp, minimum identity = 70%, and minimum alignments = 50). The degree of similarity of two genomes (*e*.*g*. a draft genome and reference genome) and the relative position (or scaffolding) of each contig within the genome was determined and visualized using two open-source alignment programs—the Contig Assembly of Prokaryotic Draft Genomes using Rearrangements (CAR) [45] and Mauve [46].

Sequence and function based genome comparisons were also performed using RAST [36]. All eleven genomes were uploaded to the RAST server. To determine the degree of similarity between two genomes at the protein level, the average amino acid identity (AAI) was calculated. Using the output from the RAST sequence-based comparison tool, the bidirectional best-hit proteins were uploaded to the online AAIr calculator (http://lycofs01.lycoming.edu/~newman/AAI/).

The core set of genes common to the eleven sequenced *Sulfurospirillum* was examined and comparisons between two clustering algorithms, COGtriangles [47] and orthoMCL [48], generated a total of 6,246 clusters of orthologous sequences that were used for downstream pan-genome analysis with GET_HOMOLOGUES software package (Fig F in S1 File.) [49]. The pan-genome was partitioned into compartments (e.g. core, soft core, cloud and shell) based upon the frequency of each gene cluster across each genome (i.e. core = 11, soft core = ≥ 10, shell = 3–9, and cloud = ≤ 2). Clusters of orthologous sequences described here for the pan-genome analysis are defined as homologous gene families found at the intersection of COGtriangles and OrthoMCL algorithms. The complete pan-genome analyses for the 11 *Sulfurospirillum* proteomes examined herein are contained in S2 File.

The parsimony pan-genome tree was constructed using the GET_HOMOLOGUES software package [49], following the user manual. As stated above, the homologous gene families found at the intersection of COGtriangles and OrthoMCL algorithms were utilized as input to obtain a pan-genome matrix, which was produced in MEGA [39] with a midpoint root.

Comparative genome analyses were performed by first finding genes of interest in RAST based upon the protein function the gene encodes for (*e*.*g*. nitrous-oxide reductase). The gene was then searched against the nr protein database using the PSI-BLAST tool in RAST (default settings; inclusion threshold = 0.002; cutoff score = 10) (*e*.*g*. top hit for the nitrous-oxide reductase was ‘cytochrome C [*Sulfurospirillum* sp. MES], max score = 1769, Query coverage = 100%, e-value = 0, Identity = 100%, Accession = gi|72890371). If a protein was not found in the nr protein database, as was the case for a few of the draft genomes that have not yet been annotated using the NCBI pipeline, the amino acid sequence from *S*. *cavolei* MES or *S*. *multivorans* was used to search the genome of interest in RAST (using BLASTP 2.2.26; cutoff score = 10). The *S*. *multivorans* genome was recently curated manually by Goris and coworkers [17], and thus served as a template to look for homologues in other *Sulfurospirillum* genomes, especially the molybdopterin oxidoreductases (Table K in S1 File). Specifically, each individual protein from *S*. *multivorans* was searched against the 10 other genomes in RAST using the aforementioned BLAST settings in RAST, and the top hits were reported. A protein was deemed present if it had a sequence identity > 20% spanning > 50% of the sequence, and in the case for multi-subunit proteins, found sequentially in the genome.

NosZ and FeFe hydrogenase phylogeny: To examine proteins closely related to NosZ from *S*. *cavolei* MES a BLASTp analysis was performed (default settings). Further phylogenetic analysis was performed on the top hits (*e*.*g*. having at least 50% identity and a 90% query coverage) to the query amino acid sequence using a neighbor-joining tree constructed using a Poisson model with a bootstrap value of 1000.

## Results and Discussion

### Phylogenomic Analysis of *Sulfurospirillum cavolei* MES

**Fig 1:**
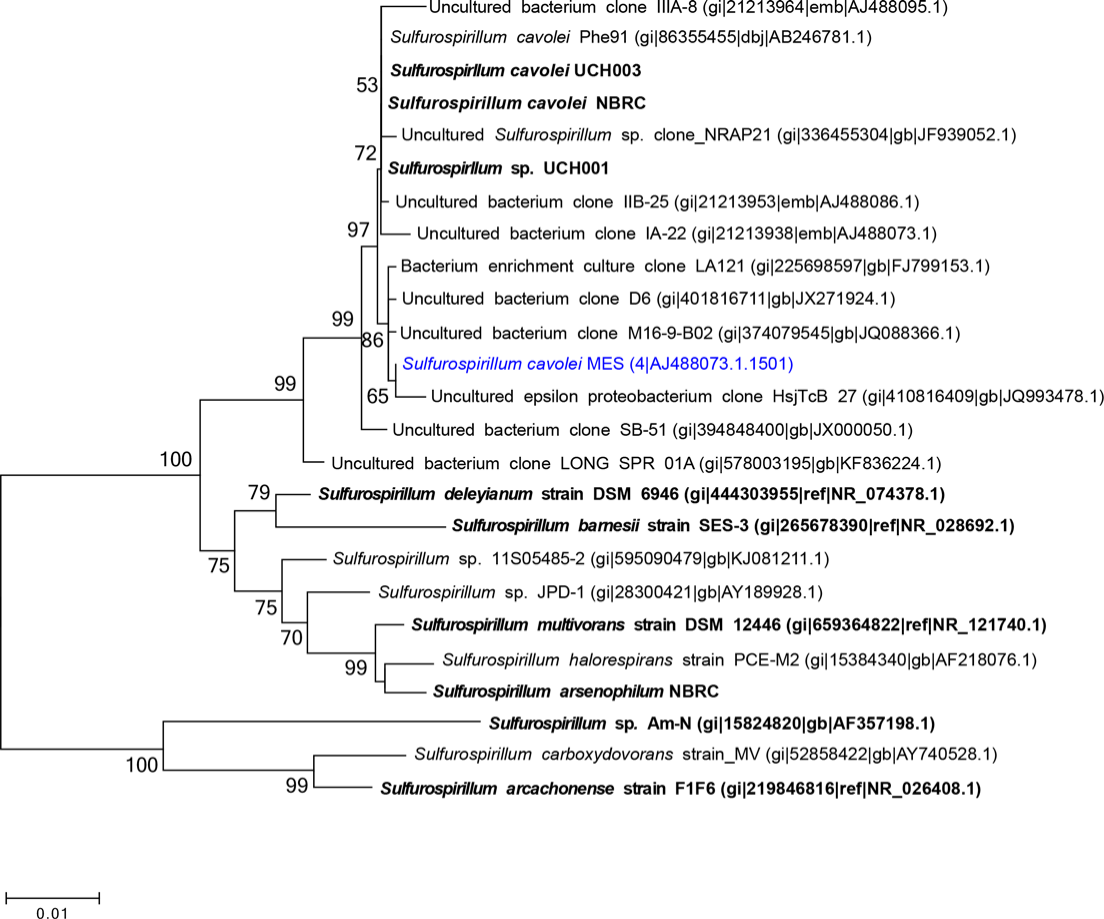
Phylogenetic tree of *Sulfurospirillum*. The unrooted tree was constructed using the Neighbor-Joining method with near complete 16S rRNA gene sequences, with a bootstrap value of 1000. Distance bar represents one substitution per 100 nucleotide positions. Strains with sequenced genomes (draft or complete) are denoted in bold. *S*. *cavolei* MES is highlighted in blue. The accession numbers for the 16S rRNA gene from *S*. *cavolei* NBRC were not publicly available and were alternatively identified by BLASTn analysis of the *S*. *cavolei* genome using the 16S rRNA gene from *S*. *cavolei* MES as query.

Phylogenetic analysis of the near full-length 16S rRNA gene grouped *S*. *cavolei* MES with *Sulfurospirillum cavolei* NBRC as the closest characterized and sequenced strain, (Fig 1). Several of the closest relatives based on 16S were uncultured clones from crude oil reservoirs [50]. Outside of the *S*. *cavolei* group, other *Sulfurospirillum* species cluster closely (*S*. *deleyianum* and *S*. *barnesii*; *S*. *multivorans*, *S*. *halorespirans*, and *S*. *arsenophilum*) but a more distantly related group consisting of *S*. sp. Am-N and *S*. *arcachonense* emerged (Fig 1).

To gain further insight into the evolutionary relatedness of the *Sulfurospirillum* genetic repertoire beyond the 16S rRNA gene, a combination of alternative whole genome analyses, including digital DNA-DNA hybridization (DDH), average nucleotide identity (ANI), and average amino acid identity (AAI) were utilized. The draft genome sequence of *Sulfurospirillum cavolei* MES [11] was compared to all other currently publically available sequenced *Sulfurospirillum* strains (Table 1). Estimates for GLM-based digital DNA-DNA hybridization (DDH) between *Sulfurospirillum cavolei* MES (present study) and *S*. *multivorans*, *S*. *deleyianum*, *S*. *barnesii*, *S*. strain Am-N, *S*. sp. SCADC, *S*. *arcachonense*, or *S*. *arsenophilum* genome sequences ranged from 17% to 20%, well below the 70% species cutoff [44,51] and were similar to the 14% DNA-DNA hybridization value previously reported for *S*. *cavolei* strain Phe91 and *S*. *deleyianum* [25]. A comparison of *Sulfurospirillum cavolei* MES and *S*. *cavolei* NBRC, or *S*. *cavolei* UCH003 revealed DDH values above 80%, support for *S*. *cavolei* MES and *S*. *cavolei* being the same species. The ANI was calculated using both best hits and reciprocal best hits between two genomes [42]. Values ranged from 77.36% to 96.74% for *Sulfurospirillum cavolei* MES and *S*. *arcachonense* or *Sulfurospirillum cavolei* MES and *S*. *cavolei* NBRC, respectively (Table D in S1 File) [15]. Similar to ANI, AAI values ranged from 61.60 between *Sulfurospirillum cavolei* MES and *S*. *arcachonense* to 97.01 for *Sulfurospirillum cavolei* MES and *S*. *cavolei* NBRC. Both AAI and ANI between *S*. *cavolei* MES and both *S*. *cavolei* strains (UCH003 and NBRC) were above 95% species cutoff (Table D in S1 File) [52]. Taken together, the draft *Sulfurospirillum cavolei* MES genome (JSEC00000000) described here is closely related to *Sulfurospirillum cavolei* strain NBRC and results indicate *S*. *cavolei* MES is a new *Sulfurospirillum cavolei* strain. Therefore, as stated previously, we suggest the strain designation of *Sulfurospirillum* sp. strain MES [11] be modified to reflect recent findings and therefore classified into species *Sulfurospirillum cavolei*.

To visualize the genome organization and gene synteny, scaffolds from *Sulfurospirillum cavolei* MES were mapped to the existing complete *Sulfurospirillum* genomes of *S*. *barnesii*, *S*. *deleyianum*, *S*. *multivorans*, *S*. *cavolei* NBRC or *S*. *cavolei* UCH003. The greatest number of contigs (and basepairs) mapped to the *S*. *cavolei* UCH003 genome (41 contigs; 2,608,390 bp), followed by the genomes of *S*. *cavolei* NBRC genome (38 contigs; 2,494,081 bp), *S*. *multivorans* (31 contigs; 2,554,942 bp), *S*. *deleyianum* (12 contigs; 2,002,475 bp) and *S*. *barnesii* (12 contigs; 1,886,367 bp), further support that *Sulfurospirillum cavolei* MES is most closely related to *S*. *cavolei*. The relative location of each *S*. *cavolei* MES scaffold was determined via alignment with the *S*. *cavolei* UCH003 complete genome (Figs B-D in S1 File).

### *Sulfurospirillum* Pan-genome Analysis

**Fig 2:**
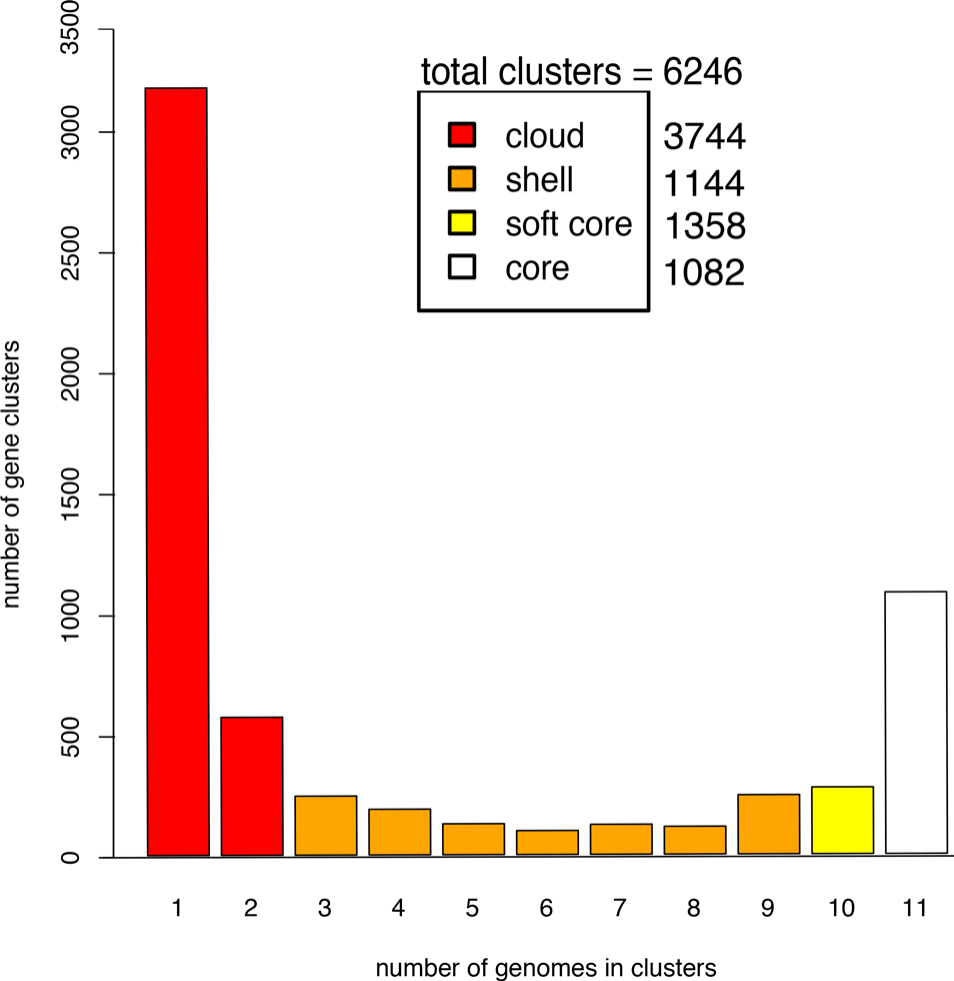
Pan-genome analysis of 11 *Sulfurospirillum* genomes. Pan-genome matrix partitioned into core, soft core, shell, and cloud components (core = all 11 species, soft core = ≥ 10, shell = 3–9, and cloud = ≤ 2).

In order to establish potential metabolic commonalities with the *Sulfurospirillum* genus, metabolic reconstruction was performed for functional genome comparisons [53]. Furthermore, to establish a common core genome, putative genes common across all eleven genomes were examined. The pan-genome is defined as the total genes in a pre-defined group [54,55]. A core genome, or repertoire of genes common across all members of a pre-defined group, can be extracted from the pan-genome. Pan-genome analysis of eleven sequenced *Sulfurospirillum* yielded insight into estimates of core genome and pan-genome sizes (Fig E in S1 File). The pan-genome had a total of 6246 clusters of orthologous sequences across 11 taxa (Fig 2). Using this method the core genome was estimated to contain 1082 clusters of orthologous sequences including an assortment of ABC transporters, molybdopterin oxidoreductases and molybdenum cofactor biosynthesis proteins, chemotaxis and flagellar machinery, and cytochrome *c* oxidases, consistent with the metabolic and ecological diversity of the *Sulfurospirillum* genus. Interestingly, close to 17% of the core genome was comprised of hypothetical proteins, suggesting much of the common functionality of this genus is currently undefined. The soft core, shell, and cloud genome contained 1358, 1144, and 3744 clusters of orthologous sequences representing 21.7%, 18.3%, and 59.9% of the total clusters, respectively. Gene clusters found in only a single genome represented 51.5% of the total clusters of orthologous sequences present in the pan-genome. A recent pan-genome analysis of 39 members of the class *Epsilonproteobacteria* revealed the core genome comprised of 15% of all the genes in an average epsilonproteobacterial genome and 67% occurred only in a single genome [56]. These findings suggest the metabolic diversity found within the *Epsilonproteobacteria* is retained within the *Sulfurospirillum* genus. Furthermore, similar values were observed for core (22%) and strain-specific (48%) protein-coding sequences (CDSs) for the pan-genome (a total of 9782 CDSs) of 10 *Shewanella* isolates [57], an environmentally relevant group of *Proteobacteria*.

**Fig 3:**
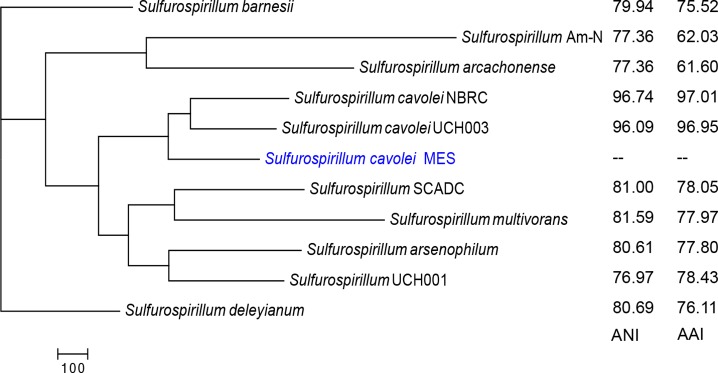
Parsimony pan-genome tree for eleven *Sulfurospirillum* proteomes. Phylogeny is based upon the gene family content (presence or absence of homologous genes) of each proteome, and the parsimony pan-genome tree was constructed with a midpoint root. *S*. *cavolei* MES is denoted in blue. Inset: Next to each organism is the average nucleotide identity (two way ANI) and average amino acid identity (AAI) compared to *S*. *cavolei* MES.

To gain a deeper understanding of the relationship among *Sulfurospirillum* species, proteomic data derived from predicted gene translation of all eleven *Sulfurospirillum* were analyzed [49]. A parsimony pan-genome tree was generated based upon the presence of homologous genes of each predicted proteome (Fig 3). According to the parsimony pan-genome tree, *S*. *cavolei* MES was most closely related to *S*. *cavolei* NBRC. This is in agreement with results from average nucleotide identity and average amino acid identity calculations (Fig 3 inset; Table D in S1 File). Furthermore, the parsimony pan-genome tree corroborates the 16S phylogeny with similar clustering of *S*. *deleyianum* with *S*. *barnesii*, and *S*. sp. Am-N and *S*. *arcachonense*. Results are consistent with *Sulfurospirillum* spp. grouping by environmental setting and metabolism, not geographical location [9], *e*.*g*. *S*. *deleyianum* and *S*. *barnesii* originated from similar freshwater habitats, while *S*. sp. Am-N and *S*. *arcachonense* were isolated from marine sediment.

### Shared Predicted Genome-based Metabolism

The shared predicted genome-based metabolism represents the gene(s) and/or pathways present in at least two genomes examined. Comparison of the eleven *Sulfurospirillum* proteomes revealed similar distributions of subsystem feature counts (*i*.*e*. RNA metabolism, sulfur metabolism, nitrogen metabolism) (Fig E in S1 File). This distribution was common to *Epsilonproteobacteria* outside of the *Sulfurospirillum* genus (*e*.*g*. *Campylobacter curvus*) but not in other proteobacteria (*e*.*g*. *E*. *coli* ATCC 8739). Inferred metabolic capabilities of *S*. *cavolei* MES are discussed below with comparisons to the ten other sequenced *Sulfurospirillum*. Specifically, pathways involved in central carbon metabolism, nitrogen metabolism, sulfur metabolism, and alternative terminal respiratory reductases are detailed below.

#### Central carbon metabolism

The eleven sequenced *Sulfurospirillum* genomes contain a complete TCA cycle (Fig G and Table E in S1 File). The glyoxylate bypass is presumably not active in any strain since isocitrate lyase was not detected in any genome and only *S*. *arcachonense*, *S*. *multivorans*, *S*. SCADC, and the three *S*. *cavolei* strains (MES, NBRC, and UCH003) encode for a malate synthase (*S*. *cavolei* MES: OA34_00390). All eleven genomes contain the class I fumarate hydratase while *S*. *arcachonense*, *S*. *barnesii*, *S*. *multivorans*, *S*. SCADC, *S*. *arsenophilum*, and *S*. sp. UCH001 also encode for the class II fumarate hydratase, which is known to be thermally stable and have no iron requirements [58]. All strains contain pyruvate carboxylase subunit B (EC 6.4.1.1) while *S*. *cavolei* MES and *S*. *deleyianum* also encode for pyruvate carboxylase subunit A, suggesting the ability to convert pyruvate into oxaloacetate via this pathway. With the exception of *S*. *deleyianum*, all of the sequenced *Sulfurospirillum* are capable of utilizing lactate as an electron donor [20,23,25]. The phosphate acetyltransferase-acetate kinase pathway was present in all strains, suggesting the ability to convert acetate into acetyl-CoA.

The classic glycolysis-TCA cycle does not seem to be operative in the *Sulfurospirillum spp*. examined here, and Acetyl-CoA may be fed into the TCA cycle from lactate or acetate, as all *Sulfurospirillum* genomes examined here encode for the ability to use lactate (except *S*. *deleyianum*) and acetate. It does not appear that more complex carbon sources can be utilized, indicating a specific niche for the degradation of fermentation products coupled to anaerobic respiration of various TEAs. The TCA cycle could alternatively be utilized for CO_2_ fixation or biosynthetic purposes. In order for the TCA cycle to operate in reverse, three key enzymes are required—ATP citrate lyase, 2-oxoglutarate:ferredoxin oxidoreductase, and fumarate reductase. All genomes were found to encode for 2-oxoglutarate:ferredoxin oxidoreductase and fumarate reductase but only *S*. SCADC (JU57_09310–09315) and *S*. *multivorans* (SMUL_0066–0067) encode for an ATP citrate lyase. While a few of the genomes encode for the reductive TCA cycle (rTCA), to our knowledge no experimental evidence has proven *Sulfurospirillum* can fix CO_2_ via the rTCA cycle. If the reductive TCA cycle is operative in *Sulfurospirillum*, then it is likely that the fixed carbon generated via this pathway is shunted through gluconeogenesis, similar to what is observed in other *Epsilonproteobacteria* [59].

Common amongst all sequenced strains was the non-oxidative phase of the pentose phosphate pathway, which provides glycolytic C6 intermediates from C5 sugars. The full Embden-Meyerhof pathway was not found in any of the eleven *Sulfurospirillum* genomes. Rather, all strains examined encode for the glycolysis-core module with conversion of dihydroxyacetone phosphate (DHAP) to pyruvate. All genomes examined contain the non-phosphorylative Entner-Duodoroff pathway, encoding for the ability to convert glycerate to pyruvate. The pathway includes glycerate kinase (*S*. *cavolei* MES: OA34_11740), which converts D-glycerate into 3-phospho-D-glycerate, 2,3-bisphosphoglycerate-independent phosphoglycerate mutase (*S*. *cavolei* MES: OA34_12420), enolase (*S*. *cavolei* MES: OA34_11835), and pyruvate kinase (*S*. *cavolei* MES: OA34_02705).

Linking the oxidation of organic substrates to the electron transport chain occurs via respiratory complex I (Table J in S1 File). *S*. *multivorans* encodes for two types of NADH:quinone oxidoreductase-like complexes; an ε-proteobacterial type complex (SMUL_195–208), which may link pyruvate oxidation to the electron transport chain using ferredoxin/flavodoxin as an electron carrier, and a complex resembling *nuoEF* from *E*. *coli* (SMUL_508–521), which may link multiple dehydrogenases to the electron transport chain [17]. All eleven genomes examined here contain the ε-proteobacterial type complex (ε-NADH I), while the only a subset encode for the second type (NADH I).

#### Nitrogen metabolism

Microbial nitrogen metabolism plays an important role in inorganic nitrogen cycles, wastewater treatment, and transformation of environmental pollutants [60]. The *S*. *cavolei* MES draft genome encodes for the respiratory periplasmic nitrate reductase (Nap) (Fig H, Table G, and Table J in S1 File)[61,62]. The gene content and organization of the *napAGHBFLD* cluster in *S*. *cavolei* MES was identical to what is commonly found in other *Epsilonproteobacteria* (e.g. *Wolinella* and other *Sulfurospirillum* spp.)[61,62]. Physiological studies have shown *S*. *cavolei* strain Phe91 and *S*. *multivorans* reduce nitrate to nitrite [1,2,22,25], while *S*. *deleyianum*, *S*. *barnesii*, *S*. *arsenophilum*, and *S*. *halorespirans* reduce nitrate completely to ammonium [1,19–21]. The *S*. *cavolei* MES genome also encodes for the nitrite reductase (Table J in S1 File), and while physiological data are needed for confirmation, the presence of both the nitrate reductase (*nap*), and nitrite reductase (*nrf*) operons suggest nitrate ammonification in this organism.

Denitrification involves four enzyme complexes (*e*.*g*., nitrate reductase, nitrite reductase, nitric oxide reductase, and nitrous oxide reductase). As stated above, all *Sulfurospirillum* strains examined contain a nitrate reductase. Nitric oxide reductase catalyzes the reduction of nitric oxide to nitrous oxide and is encoded by the *nor* operon [63]. The only sequenced *Sulfurospirillum* genome with the *nor* gene cluster was *S*. strain Am-N, which encodes for NorD, NorE, NorQ, and the B/C subunit of the nitric oxide reductase. NorBC is a cNOR that utilizes reduced cytochrome *c* as reductant [63]. This cluster shares sequence similarity with *Sulfurovum* sp. FS06-10 (PATRIC Genome ID: 1539064.3), which was isolated from a sulfidic fissure spring in Italy.

The next step in denitrification is nitrous oxide reduction, with nitrous oxide reductase catalyzing the two-electron reduction of nitrous oxide to nitrogen using reduced cytochrome *c* [64]. The *S*. *cavolei* MES draft genome encodes for two copies of the nitrous-oxide reductase protein NosZ (OA34_09370 and OA34_10415) and one copy of the accessory maturation proteins NosD (OA34_10405), NosF (OA34_10380), NosL (OA34_10375) and NosY (OA34_10345).

**Fig 4:**
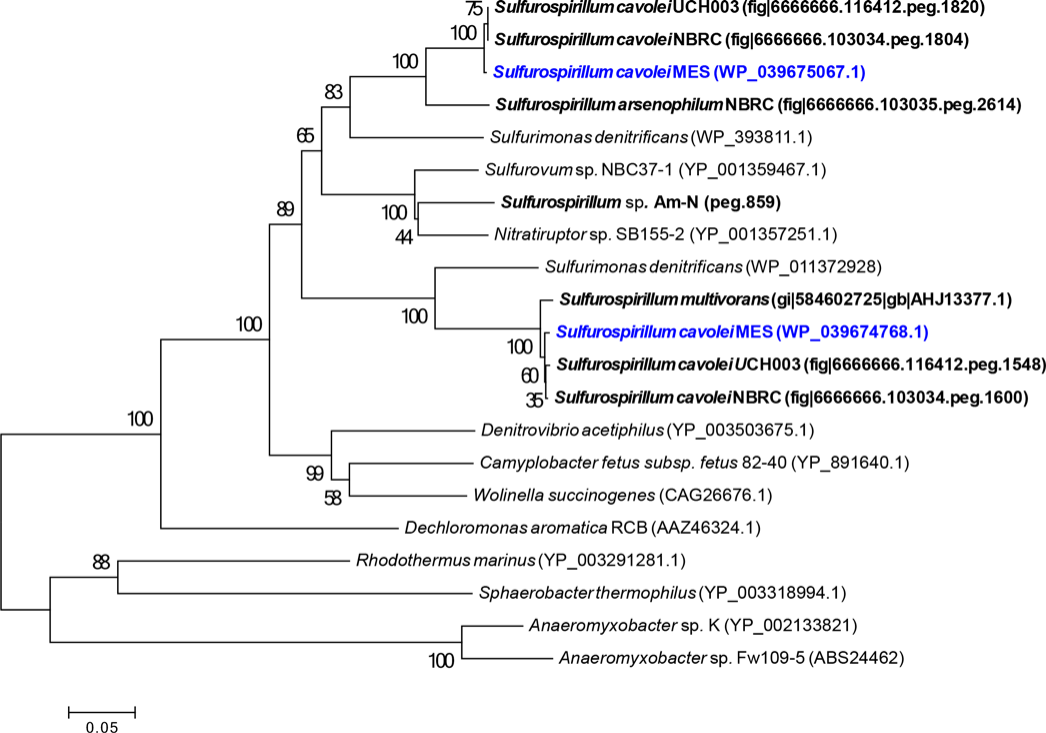
Neighbor-joining tree of the NosZ amino acid sequence. Bootstrap value of 1000. *S*. *cavolei* MES is denoted in blue.

Phylogenetic analysis of NosZ amino acid sequences revealed two separate *Sulfurospirillum* clades of nitrous-oxide reductases (Fig 4). Both clades were found within a branch containing full length epsilonproteobacterial NosZ sequences with the characteristic C-terminal cupredoxin domain (CuA) and *c-*heme (-CXXCH-) domain, similar to the nitrous-oxide reductase from *W*. *succinogens* [65–67]. One clade contained the full-length NosZ protein sequences from *S*. *cavolei* MES (OA34_09370), *S*. *multivorans*, *S*. *cavolei* NBRC, and *S*. *cavolei* UCH003 that contain no adjacent *nos* accessory proteins. Interestingly, the *nosZ* gene from *S*. *multivorans* (SMUL_2124) was found between two transposase IS4 genes (SMUL_2120 and SMUL_2126), and Goris and coworkers have suggested its acquisition via horizontal gene transfer [17].

**Fig 5:**
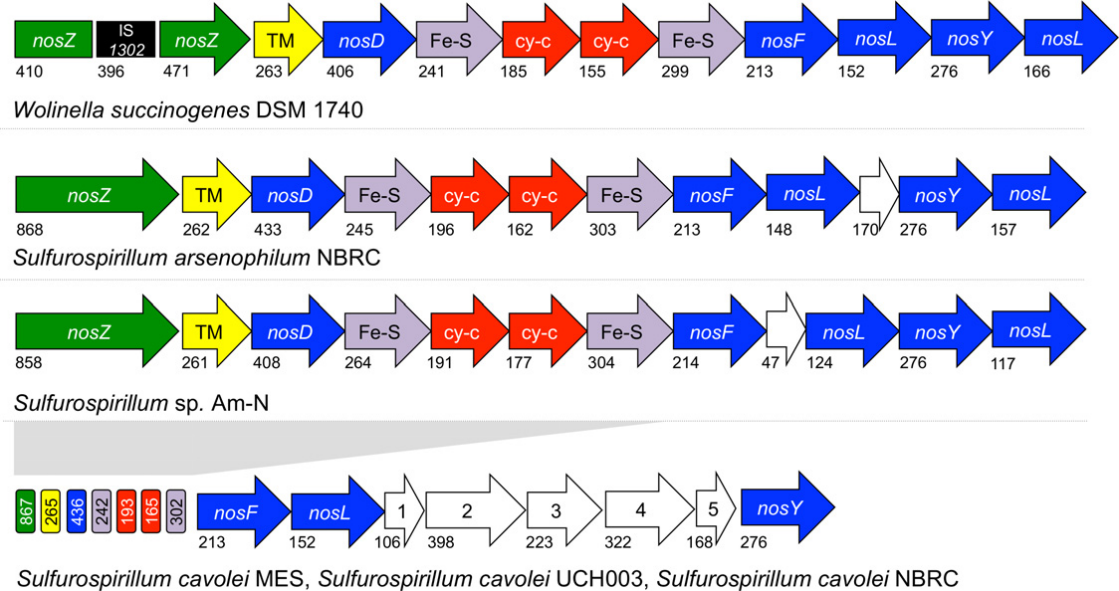
Operon organization of nitrous oxide reduction pathway. Nitrous oxide reductase gene cluster of *Wolinella succinogens* (top) and *Sulfurospirillum* spp. (bottom). Color scheme was maintained from reference [18]. Green ORFs encode for nitrous oxide reductase, yellow ORFs encode a conserved trans-membrane protein, blue ORFs encode for *nos* accessory proteins, purple ORFs encode for 4Fe-4S proteins, red ORFs encode for *c-*type cytochromes, and white ORFs (labeled 1–5) represent additional predicted genes not found in the *W*. *succinogens nos* cluster. Putative function of additional ORFs: 1. Hypothetical protein (Locus tag: OA34_06690), 2. ABC transporter permease (OA34_10365), 3. ABC transporter ATP-binding protein (OA34_10360), 4. Hypothetical protein (OA34_10355), 5. Hypothetical protein (OA34_06670). Note: ORFs not drawn to scale, the length of the predicted protein (in amino acid residues) encoded by each gene is provided.

The other clade included NosZ protein sequences from *S*. *cavolei* MES (OA34_09370), *S*. *arsenophilum* NBRC, *S*. *cavolei* NBRC, and *S*. *cavolei* UCH003 and contained the full-length NosZ protein and adjacent accessory proteins NosD, NosF, NosL, and NosY (Fig 5). The configuration of the *nos* cluster in *S*. *arsenophilum* and *S*. Am-N is almost identical to *Wolinella succinogenes*, while all three *S*. *cavolei* strains contain an identical *nos* cluster with a five-gene insertion between *nosL* and *nosY*. Two of the five genes in this cluster encode for an ABC transporter permease (*S*. *cavolei* MES: OA34_10365) and ABC transporter ATP-binding protein (*S*. *cavolei* MES: OA34_10360).

With the aforementioned *Sulfurospirillum* strains encoding for the complete *nos* cluster, the genus undoubtedly has the potential to contribute to nitrous oxide consumption and thus help mitigate the emission of this potent greenhouse gas. Recent work has shown that of the Bacteria and Archaea that contain an atypical *nos* cluster, 44% are denitrifiers, 56% contain no other nitrification genes, and 31% perform dissimilatory nitrate reduction to ammonium (DNRA). Further physiological studies are needed for confirmation of nitrous oxide reduction but these findings suggest that the three *S*. *cavolei* strains (MES, UCH003, and NBRC), *S*. *arsenophilum*, and *S*. Am-N belong with the other 31% of DNRA Bacteria and Archaea that contain an atypical *nos* cluster [18].

The ability to fix nitrogen has been shown in *S*. *multivorans* [17,68], with the nitrogenase structural genes contained in two clusters, SMUL_1286–1287 and SMUL_1656–1659 [17]. Based upon amino acid sequence homology, *S*. *cavolei* MES only encodes for the molybdenum-iron type nitrogenase (Table J in S1 File). Physiological data is needed to confirm these findings, but based upon the presence this gene cluster and synteny of the nitrogenase gene and the accessory maturation genes it is likely that *S*. *cavolei* MES is capable of nitrogen fixation via this pathway. All strains examined (with the exception of *S*. Am-N and *S*. *deleyianum*) encode for the molybdenum-iron type nitrogenase. Moreover, like *S*. *multivorans*, *S*. *cavolei* UCH003 and *S*. *cavolei* NBRC also encode for the iron-iron nitrogenase. The ability to assimilate ammonium was also evident in all strains examined, with the presence of an ammonium transporter (*S*. *cavolei* MES: OA34_00505), and the glutamine synthetase-glutamate synthase pathway.

Overall, these findings suggest that *S*. *cavolei* MES reduces nitrate to ammonium (using Nap and Nrf), utilizes a molybdenum-iron type nitrogenase for nitrogen fixation, and encodes for the glutamine synthetase-glutamate synthase pathway for nitrogen assimilation via ammonium. All 11 genomes examined encode for the ability to assimilate ammonium, a process that is especially important in oil reservoirs where ammonium is the primary source of nitrogen [69], and where *S*. *cavolei* was first isolated [15,25]. Interestingly, all three *S*. *cavolei* strains (MES, NBRC, and UCH003), *S*. *arsenophilum*, and *S*. strain Am-N encode for a nitrous oxide reductase with adjacent accessory proteins, suggesting the ability to reduce nitrous oxide and act as a biological sink for this potent greenhouse gas.

#### Sulfur metabolism

A commonality found among the *Epsilonproteobacteria* isolated to date is their involvement in the sulfur cycle. For example, most *Sulfurospirillum* are facultative anaerobic sulfur-reducing bacteria and belong to the Group 2 sulfur reducers, which consists of *Wolinella*, *Campylobacter*, and *Shewanella* [23]. To determine if *S*. *cavolei* MES also shares this capability, the draft genome was analyzed for the presence of genes/pathways specific to the sulfur cycle (Table H and Fig I in S1 File). The *S*. *cavolei* MES genome encodes for genes necessary for assimilatory reduction of sulfate to sulfite via adenylylsulfate (APS) and 3’-Phosphoadenylyl-sulfate (PAPS), and the subsequent reduction of sulfite to hydrogen sulfide (Fig I in S1 File). Comparison to other sequenced genomes revealed the presence of genes involved in inorganic sulfur assimilation in all strains. Interestingly, while the genome of *S*. *deleyianum* encodes for inorganic sulfur assimilation, *S*. *deleyianum* requires reduced sulfur for growth, suggesting this pathway is inactive [14,19]. Despite encoding for a cytochrome *c* sulfite reductase (similar to *mccA* from *Wolinella succinogenes*) (Fig I in S1 File) physiological evidence suggests *S*. *arcachonense* and *S*. *barnesii* are unable to utilize sulfite as an electron acceptor [20,23,24]. According to our findings, assimilatory sulfate reduction is common amongst the sequenced *Sulfurospirillum* species examined, with the exception of *S*. Am-N and *S*. *deleyianum*. Furthermore, all 11 genomes contained serine acetyltransferase and cysteine synthase for biosynthesis of cysteine from sulfide.

Biological thiosulfate reduction is an important process in sulfur cycling that occurs in anoxic sediments and commonly performed by sulfate reducers in these environments [70]. Physiological evidence suggests that the majority of sequenced *Sulfurospirillum* utilize thiosulfate as an electron acceptor [19,20,23,25]. This is substantiated by genome data, where all genomes examined (excluding *S*. *arcachonense* and *S*. Am-N) encode for a gene cluster with high similarity to the *S*. *multivorans* thiosulfate reductase cluster (SMUL_0346–0348), which is likely involved in thiosulfate respiration [17].

Other sulfur-containing electron acceptors utilized by *Sulfurospirillum* species include tetrathionate and polysulfide. The genomes of *S*. SCADC, *S*. sp. UCH001, and the three *S*. *cavolei strains* encode for a tetrathionate reductase with high identity to the *S*. *multivorans* gene cluster (SMUL_2568–2571). Furthermore, with the exception of *S*. Am-N, *S*. *arcachonense*, and *S*. sp. UCH001, all strains encode for a polysulfide reductase with similarity to SMUL_0342–0344 for the reduction of polysulfide for energy conservation. Generally speaking, *Epsilonproteobacteria* are able to oxidize sulfur compounds, especially in hydrothermal vents. Surprisingly, *Sulfurospirillum* SCADC is the only *Sulfurospirillum* species analyzed here that contains genes for oxidation of reduced inorganic sulfur compounds, found in a single *soxXYZABCD* operon [16,71]. The proteins encoded by this operon are most closely related to other Epsilonproteobacteria (*Arcobacter*, *Nitratifractor*, *Sulfurovum*) and Gammaproteobacteria (*Beggiatoa* and *Marinobacterium*). While the *sox* operon was found only in *S*. SCADC, it has been proposed by Goris and coworkers that *S*. *multivorans* may oxidize sulfide via a bidirectional polysulfide reductase/sulfide dehydrogenase (SMUL_3273–3275) [17]. Indeed all sequenced *Sulfurospirillum* strains examined here encode for a homologous gene cluster (*S*. *cavolei* MES: OA34_12905-OA34_12915) (Table K in S1 File), and therefore may utilize this pathway for sulfide oxidation.

Overall, the major routes of sulfur utilization in *Sulfurospirillum* are diverse and include inorganic sulfate assimilation, thiosulfate reduction, tetrathionate reduction, and polysulfide reduction: the *S*. *cavolei* MES genome encodes for all of these pathways. Interestingly, compared to their freshwater counterparts, the marine *Sulfurospirillum* isolates (*e*.*g*. *S*. *arcachonense* and *S*. Am-N) have a constrained sulfur metabolism. The *S*. *arcachonense* genome does not encode for thiosulfate, tetrathionate, or polysulfide reductase, and physiological data substantiate these findings [23,24]. While physiological data is lacking, genome data suggests *S*. Am-N is unable to utilize these sulfur compounds as terminal electron acceptors. Further physiological characterization of these pathways across all sequenced *Sulfurospirillum* strains is needed and would shed light on the impact these organisms have on sulfur cycling in environments where they reside.

#### Other respiratory reductases

*Sulfurospirillum* spp. respire using a multitude of terminal electron acceptors [2,19,20,22–24]. As such, all sequenced *Sulfurospirillum* genomes, including that of *S*. *cavolei* MES, were found to encode for numerous respiratory reductases (*e*.*g*., arsenate, DMSO, TMAO), the majority of which are molybdopterin oxidoreductases, and have been examined in detail by Goris and coworkers [17]. The *S*. *multivorans* genome encodes for an arsenate reductase (SMUL_3145–3147), DMSO reductase (SMUL_0500–0501), and TMAO reductase (SMUL_2312–2314) [17]. The ability to utilize arsenate as an electron acceptor has been observed in *S*. *cavolei*, *S*. *deleyianum*, *S*. *multivorans*, *S*. *barnesii*, *S*. *arsenophilum*, *S*. *halorespirans*, and *S*. *carboxydovorans* [2,14,20,23–25]. Only *S*. *arsenophilum*, *S*. sp. UCH001, and *S*. *barnesii* contain gene clusters with high sequence similarity (>68% identity for all three subunits) to the arsenate reductase from *S*. *multivorans* (SMUL_3145–3147). Thus, while multiple *Sulfurospirillum* strains reduce arsenate, for most strains it is unclear what genes encode for this activity (Table K in S1 File). With the exception of *S*. *arcachonense*, *S*. *barnesii*, and *S*. Am-N, all genomes examined encode for the DMSO reductase gene cluster (SMUL_0500–0501), which corroborates physiological data [2,19,20,22–24]. *S*. *barnesii* was the only strain to encode for the TMAO reductase gene cluster with high similarity to the *S*. *multivorans* cluster (SMUL_2312–2314). *S*. *deleyianum* does not encode for a gene cluster with similarity to SMUL_2312–2314, yet can utilize DMSO and TMAO as a terminal electron acceptor [19–21], which suggests the utilization of an alternative molybdopterin oxidoreductase encoding cluster.

The terminal reduction of oxygen is catalyzed by the *cbb*_3_-type cytochrome *c* oxidase. This ability has been observed in all cultured *Epsilonproteobacteria* to date [72], and has recently been examined in other *Sulfurospirillum* species [17]. The *S*. *cavolei* MES genome encodes for a *cbb*_3_-type cytochrome *c* oxidase (Table F and Fig J in S1 File) and a survey of the available *Sulfurospirillum* genomes revealed the presence of *ccoNOQP* (EC 1.9.3.1) in all strains. The *cbb*3-type cytochrome *c* oxidase was shown to be upregulated in *Shewanella oneidensis* when growing anaerobically on an electrode [73] and thus may have a role in oxidative stress, regulation of gene expression, or oxygen scavenging in anaerobic electrode-based microbial systems. Furthermore, all eleven *Sulfurospirillum* genomes examined encode for a cytochrome d ubiquinol oxidase.

In order to combat oxygen stress, all eleven genomes encode for a superoxide dismutase, and superoxide reductase. Specifically, *S*. *arcachonense* contains a nickel-dependent version, while the other genomes encode for an iron-containing enzyme. *S*. *multivorans* also encodes for a copper/zinc superoxide dismutase [17]. Furthermore, *S*. *cavolei* MES encodes for an iron-containing superoxide dismutase (SOD) (OA34_02085) with 80% amino acid identity to *S*. *multivorans* iron-containing SOD (SMUL_0529; AHJ11804.1) [17]. The *S*. *multivorans* genome encodes for catalase (SMUL_3224; AHJ14450.1) [17], and a survey of the other ten genomes suggests that only *S*. *barnesii* (Sulba_2521; AFL69788.1) and *S*. sp. SCADC (JU57_06945; KFL34263.1) encode for this protein.

These findings support physiological evidence of the utilization of oxygen as a terminal electron acceptor and the ability of *Sulfurospirillum* spp. to manage oxygen stress under microaerobic conditions [25]. It is not surprising that *S*. *cavolei* MES possesses these capabilities, since it originated from an open cistern that remained primarily anaerobic, but received brewery waste daily, leading to regular introduction of oxygen, and access to S and N compounds and a plethora of carbon sources. *S*. *cavolei* MES was enriched in an anoxic electrochemical cell but may be involved in scavenging oxygen that 1) leaked across the membrane separating the anode and cathode chambers of the MES and/or 2) was introduced during sampling.

### Unique strain-specific functional pathways

A survey of the pan-genome revealed a number of genes unique to individual species within the *Sulfurospirillum* genus *e*.*g*., nitric oxide reduction in *S*. Am-N, and sulfur oxidation in *S*. SCADC (as mentioned above). To better understand *S*. *cavolei* MES and its potential role in microbial electrosynthesis, the draft genome was closely examined for unique gene(s) and gene clusters. Two gene clusters unique to *S*. *cavolei* that may contribute to the utility of *S*. *cavolei* MES in electrosynthesis systems are a unique [FeFe] hydrogenase and a holin-like protein, CidA.

The [FeFe] hydrogenase is responsible for catalyzing reversible hydrogen oxidation and typically works in the direction of hydrogen formation [74]. *Sulfurospirillum cavolei* NBRC and *S*. *cavolei* UCH003 also encode for a [FeFe] hydrogenase with 98% and 92% identity to the *S*. *cavolei* MES [FeFe] hydrogenase large subunit (OA34_12480), respectively. This particular [FeFe] hydrogenase was unique to the three *S*. *cavolei* strains (Table I in S1 File). Interestingly, [FeFe] hydrogenases have been found in other electrosynthetic microorganisms, including *Sporomusa ovata* (SOV_3c02090; EQB26335.1). *S*. *ovata* is capable of extracting electrons from a negatively poised cathode to reduce CO_2_ to acetate [75] and while it is unknown if the iron hydrogenase plays a role within bioelectrochemical systems, it may function to ensure redox balance within the bacterial cell through reversible proton reduction or it may facilitate H_2_ production with subsequent support for electroacetogenesis.

**Fig 6:**
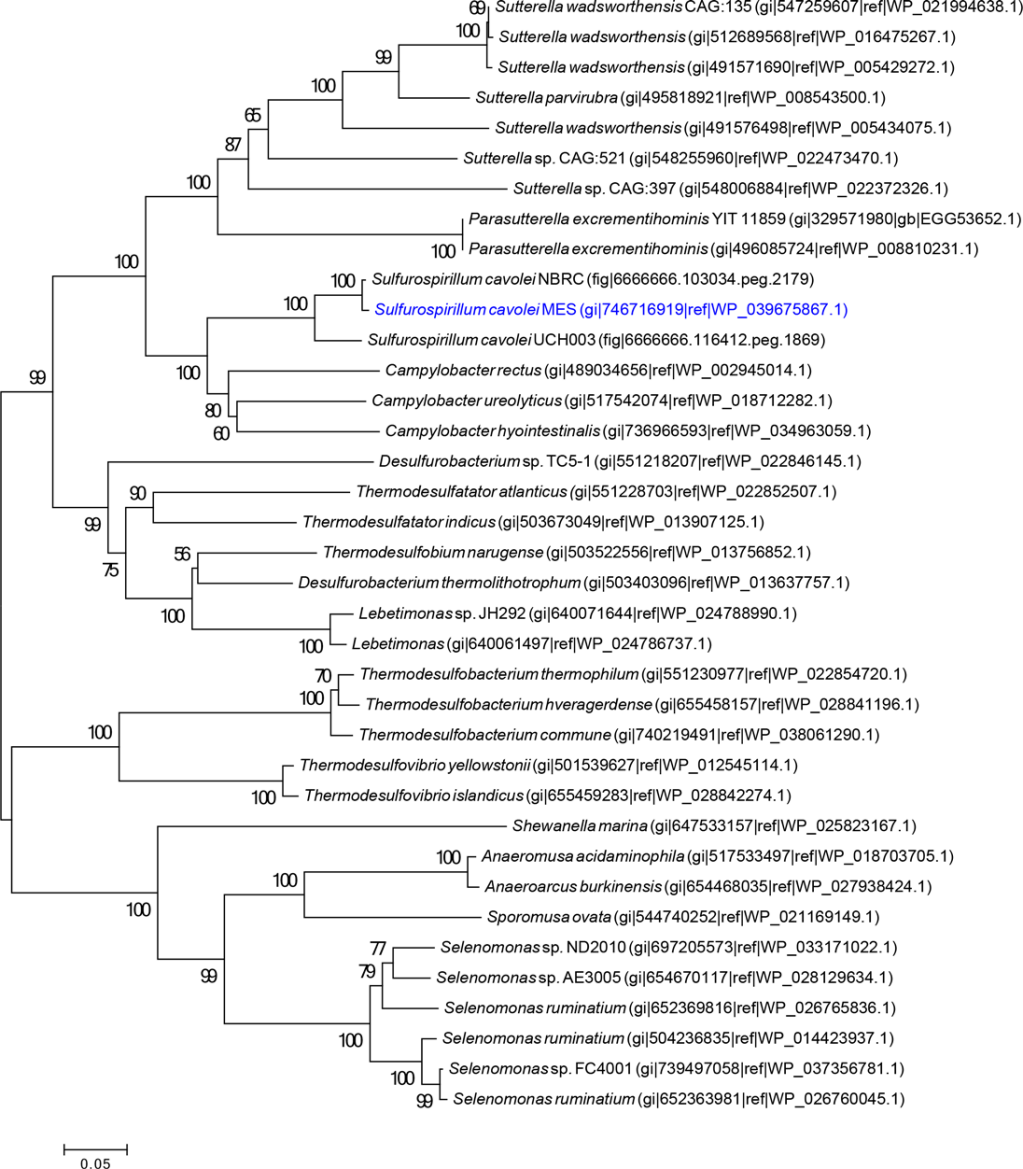
Neighbor-joining tree of *S*. *cavolei* MES iron hydrogenase. Tree consists of amino acid sequences closely related to *Sulfurospirillum cavolei* MES [FeFe] hydrogenase large subunit (denoted in blue), with a bootstrap value of 1000.

The [FeFe] hydrogenase large subunit from *S*. *cavolei* MES (OA34_12480) was most similar to other iron hydrogenases from the genus *Campylobacter*, *Sutterella*, *Thermodesulfobium*, and *Parasutterella*. Furthermore, phylogenetic analysis revealed the [FeFe] hydrogenase large subunit (OA34_12480) formed a clade with hydrogenases from *S*. *cavolei* and members of the *Campylobacter* genus (e.g. *C*. *rectus*, *C*. *ureolyticus*, and *C*. *hyointestinalis*) (Fig 6).

Maturation of iron hydrogenase proteins requires the presence of accessory proteins HydE, HydF, and HydG for proper function of the mature protein [76]. All eleven sequenced genomes encode for HydE, but only the *S*. *cavolei* MES, NBRC, and UCH003 genomes encode for all three accessory proteins (Fig J in S1 File). The gene synteny in this region also suggests complete functionality of this gene cluster. The cluster is organized with the [FeFe] hydrogenase large subunit (OA34_12480) first, followed by the [FeFe] hydrogenase small subunit (OA34_12485), [FeFe] hydrogenase membrane component (OA34_12490), HydG (OA34_12495), aspartate ammonia-lyase (OA34_12500), HydE (OA34_12505), and lastly HydF (OA34_12510) (Fig J in S1 File). Other closely related clusters contain identical gene configuration with no aspartate ammonia-lyase (*e*.*g*., *C*. *rectus*, *C*. *ureolyticus*) or with an alternative gene insertion between HydG and HydE (*e*.*g*., *S*. *oneidensis* MR-1, *S*. *halifaxensis*) (Fig J in S1 File). The significance of the aspartate ammonia-lyase in the *S*. *cavolei* [FeFe] hydrogenase gene cluster is unknown but it has been found in other [FeFe] hydrogenase gene clusters (*e*.*g*., *D*. *vulgaris*), and may play a role in linking biosynthesis and central carbon metabolism or amino acid metabolism [77,78].

Another gene of interest in the *Sulfurospirillum cavolei* MES genome was a holin-like protein CidA. CidA is part of a gene cluster responsible for programmed cell death (PCD), which includes CidA, CidB, LrgA, and LrgB [79]. Specifically CidA/B is thought to oligomerize and disrupt proton motive force [79]. It has been hypothesized that this gene cluster is active under harsh environmental conditions, including poor nutrient availability [80]. Within microbial communities PCD is hypothesized to benefit the population at the expense of a few, ensuring survival [81] and promoting biofilm formation through release of genomic DNA [79,82]. The holin-like protein CidA (OA34_03435) from *S*. *cavolei* MES was most closely related to CidA from *S*. *cavolei* UCH003 (98% identity, 98% positives). Further analysis revealed the presence of CidB (OA34_03430), LrgA (OA34_11470) and LrgB (OA34_11465) in the *S*. *cavolei* MES genome. No other sequenced *Sulfurospirillum* contained all four genes with the exception of *S*. *cavolei* UCH003. Interestingly, LrgA and LrgB were found directly upstream of the *nrf* operon. The environment each strain inhabited—*S*. *cavolei* UCH003 was isolated from groundwater contaminated with chloroethenes [15], while *S*. *cavolei* MES was enriched in a microbial electrosynthesis system [11–13]—suggests both are uniquely able to adapt to dynamic and harsh environmental conditions using this system.

### Conclusions

**Fig 7:**
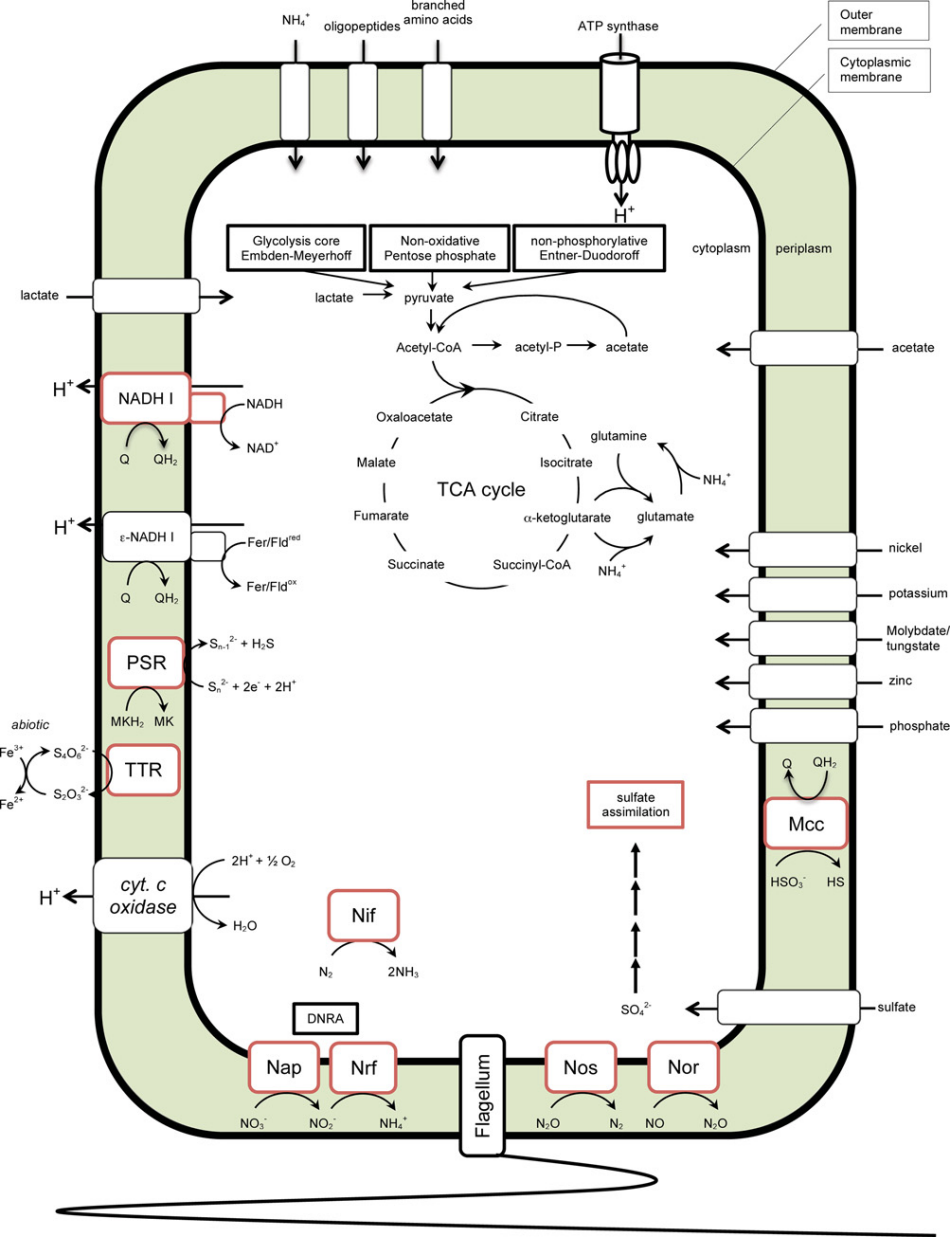
Reconstruction of central metabolism of *Sulfurospirillum* species. All enzymes or enzyme complexes depicted are found in all eleven sequenced genomes. Enzymes denoted with a red border are unique to a single genome or found in a subset of genomes. DNRA, dissimilatory nitrate reduction to ammonium; Nap, nitrate reductase; Nrf, nitrite reductase; Nif, nitrogen fixation; Nos, nitrous oxide reductase; Nor, nitric oxide reductase; cyt. *c* oxidase, cytochrome *c* oxidase; NADH-I, NADH-quinone oxidoreductase; ε-NADH I, ferredoxin/flavodoxin-quinone oxidoreductase; TTR, tetrathionate reductase; PSR, polysulfide reductase; Mcc, respiratory sulfite reductase Q, quinone; MK, menaquinone.

Here we present a detailed comparative analysis of a draft genome obtained from the metagenome of a mixed microbial community isolated from a microbial electrosynthesis system. Based upon 16S phylogeny and whole genome phylogenomics the assembled draft genome was most closely related to *Sulfurospirillum cavolei*. The *S*. *cavolei* MES draft genome was compared to ten sequenced *Sulfurospirillum* genomes (5 complete and 5 draft). Pan-genome analysis revealed a core genome common across all eleven strains examined. Metabolic comparisons and in-depth analysis of unique genes highlight potential ecological niche-specific capabilities (*e*.*g*. reductive dehalogenation, sulfur oxidation, nitrous oxide reduction) (Fig 7). Importantly, the *Sulfurospirillum* strains containing the complete nitrous oxide reduction (*nos*) pathway inhabit ecologically diverse freshwater and marine environments and represent potential nitrous oxide sinks in these settings. In addition, two unique gene clusters were found in the three *S*. *cavolei* strains, one encoding an iron hydrogenase and the other encoding genes involved in programmed cell death.

The finding that *Sulfurospirillum cavolei* MES was consistently abundant in microbial electrosynthesis systems, combined with insights from the aforementioned comparative genomic analysis, yields insight to the potential role this bacterium may play within these systems and the factors contributing to its persistence. One hypothesis for the role of *Sulfurospirillum cavolei* MES in the electrosynthesis system is that it may use biocathode-generated hydrogen as an electron donor and acetate as a carbon source. *Sulfurospirillum cavolei* strain Phe91 can utilize formate or hydrogen as electron donors in the presence of acetate [25]. If so, removal of *Sulfurospirillum* would enhance product formation. Indeed, this has been observed by LaBelle and coworkers, where the community structure shifted upon repeated exposure to acidic pH, with *Acetobacterium* as the predominant species [83]. Concomitant with this community shift was an enhancement of acetate (5–15 mM/day) and hydrogen (>1000 mM/day) compared to previous production rates [12,13,83]. *Sulfurospirillum* spp. are sensitive to pH, with *S*. *cavolei* able to tolerate a pH range of 6.0–8.0 [25]. Therefore the acidic pH likely contributed to the decrease of *Sulfurospirillum* abundance and emergence of *Acetobacterium* as the dominant species.

Another possibility is that *Sulfurospirillum* plays a role as an oxygen scavenger, consistent with its abundance in the supernatant, a likely place for introduction of oxygen into the system. Acetogens are sensitive to low concentrations of oxygen and while enzymes of the acetyl-CoA pathway are extremely sensitive to oxygen, acetate is still synthesized via this pathway in the presence of low oxygen concentrations [84]. In some cases low levels of oxygen enhanced acetate production. Thus it may be possible to regulate the acetate and hydrogen levels by controlling the abundance of *S*. *cavolei* MES and in turn contribute to the stability and longevity of the mixed community biocatalyst. Further work is needed to ascertain the role of *S*. *cavolei* MES within the microbial electrosynthesis system and to this end, metatranscriptomic analyses are currently underway to quantitate the expression of functionally important pathways specific to *Sulfurospirillum*.

## Supporting Information

S1 File **Fig A**. QUAST quality assessment of SPAdes genome assembly. (A) Cumulative length of contigs, (B) GC content, and (C) fold genome coverage. **Fig B**. Whole genome alignment of *S*. *cavolei* UCH003 with A) *S*. *cavolei* MES or B) *S*. *cavolei* NBRC. Alignments were completed with progressive Mauve (ref) and Contiguator (ref). C) Whole genome alignment of *S*. *cavolei* UCH003 with *S*. *cavolei* NBRC and *S*. *cavolei* MES. Contigs for NBRC or MES were aligned to UCH003, concatenated, and aligned with progressive Mauve. See materials and methods for details on alignments. **Fig C**. Dot plot before (A, C) or after (B, D) contig re-arrangement between *S*. *cavolei* UCH003 and *S*. *cavolei* MES (A, B) or *S*. *cavolei* NBRC (C, D). **Fig D**. Whole genome alignment between *S*. *cavolei* MES (draft) and (A and B) *S*. *multivorans* (reference), (B and C) *S*. *barnesii* (reference), and (E and F) *S*. *deleyianum* (reference) before (A, C, and E) and after (B, D, and F) contig re-arrangement with CAR. Red dots correspond to forward matches while blue dots represent reverse matches. **Fig E**. Heatmap of Subsystems categories for eleven *Sulfurospirillum* proteomes. *Campylobacter curvus* was used for comparison to a non-*Sulfurospirillum Epsilonproteobacterium*. Subsystem counts were normalized to total counts per genome. The scale from blue to red represents the Subsystems counts within each category as a percentage of the entire Subsystems counts per genome ranging from 0 to 15%.**Fig F**. (A) Core-genome and B) pan-genome size estimations as a function of the number of genomes (from 1 to 11). **Fig G**. KEGG pathway for the TCA cycle in *S*. *cavolei* MES. **Fig H**. KEGG pathway for nitrogen metabolism in *S*. *cavolei* MES. **Fig I**. KEGG pathway for sulfur metabolism in *S*. *cavolei* MES. The assimilatory sulfate reduction pathway is shown in detail with available Genbank protein IDs. The RAST idendifier and sequence identity, positives, and e-value [based upon BLASTP results against Sat, CysN, CysD, CysH or Sir from *S*. *multivorans* or MccA (UniProt:Q7MSJ8) from *Wolinella succinogens*] are shown when Genbank IDs were unavailable. **Fig J**. Comparison of gene synteny of the [FeFe] hydrogenase from *S*. *cavolei* MES, *S*. *cavolei* NBRC, and *S*. *cavolei* UCH003. Numbers represent the length of each predicted translated protein (in amino acids). **Table A**. Analysis of raw read files from PacBio and Illumina metagenome sequencing using PRINSEQ. **Table B**. Assembly statistics from various genome assembly attempts for *Sulfurospirillum* sp. strain MES. Predicted genes were determined using QUAST (A). Multiple gene prediction tools were used for the final draft genome assembly (B). **Table C**. Genome comparison within the family *Campylobacteraceae*. Data was compiled from the NCBI database [85]. **Table D**. Average nucleotide identity and average amino acid identity between *S*. *cavolei* MES and 10 other sequenced *Sulfurospirillum* complete and draft genomes. **Table E**. Annotated genes involved in carbohydrate metabolism for *S*. *cavolei* MES. **Table F**. Annotated genes having specific roles in respiration in *S*. *cavolei* MES. **Table G**. Annotated genes involved in nitrogen metabolism for *S*. *cavolei* MES. **Table H**. Annotated genes involved in sulfur metabolism for *S*. *cavolei* MES. **Table I**. Protein BLAST analysis of the iron hydrogenase from *Sulfurospirillum cavolei* MES. The protein sequence from *S*. *cavolei* MES was used to search the other 10 *Sulfurospirillum* genomes for the presence of homologous proteins. **Table J**. Manual genome curation with *S*. *multivorans*. **Table K. Comparison of molybdopterin oxidoreductases across all sequenced *Sulfurospirillum* species**. Protein sequences from *S*. *multivorans* were identified and BLASTed against each genome. The RAST identifiers along with similarity statistics are shown. **Table L. Assessing the completeness of the *Sulfurospirillum cavolei* MES draft genome**. A total of 397 marker genes were used to determine the completeness of the draft genome, with 390 found once in the draft genome and 7 found twice.
(ZIP)

S2 File Pangenome output file from GET_HOMOLOGUES. (GZ)
