# Supplementary material for: Comparative Genomic Analysis of Sulfurospirillum cavolei MES Reconstructed from the Metagenome of an Electrosynthetic Microbiome
Source: PLoS One. 2016 Mar 16;11(3):e0151214. doi: 10.1371/journal.pone.0151214 (PMC4794192; doi:10.1371/journal.pone.0151214)
Supplement: S1 File — Fig A. QUAST quality assessment of SPAdes genome assembly. (A) Cumulative length of contigs, (B) GC content, and (C) fold genome coverage. Fig B. Whole genome alignment of S. cavolei UCH003 with A) S. cavolei MES or B) S. cavolei NBRC. Alignments were completed with progressive Mauve (ref) and Contiguator (ref). C) Whole genome alignment of S. cavolei UCH003 with S. cavolei NBRC and S. cavolei MES. Contigs for NBRC or MES were aligned to UCH003, concatenated, and aligned with progressive Mauve. See materials and methods for details on alignments. Fig C. Dot plot before (A, C) or after (B, D) contig re-arrangement between S. cavolei UCH003 and S. cavolei MES (A, B) or S. cavolei NBRC (C, D). Fig D. Whole genome alignment between S. cavolei MES (draft) and (A and B) S. multivorans (reference), (B and C) S. barnesii (reference), and (E and F) S. deleyianum (reference) before (A, C, and E) and after (B, D, and F) contig re-arrangement with CAR. Red dots correspond to forward matches while blue dots represent reverse matches. Fig E. Heatmap of Subsystems categories for eleven Sulfurospirillum proteomes. Campylobacter curvus was used for comparison to a non-Sulfurospirillum Epsilonproteobacterium. Subsystem counts were normalized to total counts per genome. The scale from blue to red represents the Subsystems counts within each category as a percentage of the entire Subsystems counts per genome ranging from 0 to 15%.Fig F. (A) Core-genome and B) pan-genome size estimations as a function of the number of genomes (from 1 to 11). Fig G. KEGG pathway for the TCA cycle in S. cavolei MES. Fig H. KEGG pathway for nitrogen metabolism in S. cavolei MES. Fig I. KEGG pathway for sulfur metabolism in S. cavolei MES. The assimilatory sulfate reduction pathway is shown in detail with available Genbank protein IDs. The RAST idendifier and sequence identity, positives, and e-value [based upon BLASTP results against Sat, CysN, CysD, CysH or Sir from S. multivorans or MccA (UniProt:Q7MSJ8) [file pone.0151214.s001.zip › Supplementary Table A-D; Supplementary Figure A-J.docx]

**SUPPLEMENTARY INFORMATION.**

Ross DE, Marshall CW, May HD, and Norman RS. Comparative genomic analysis of *Sulfurospirillum cavolei* MES reconstructed from the metagenome of an electrosynthetic microbiome

**Bioinformatics pipeline for sequence processing, assembly, and annotation.**

Provided below are all programs (with their respective web address) used for sequence processing, assembly, and annotation. With the exception of CLC genomics workbench, all programs were open source and readily available for download.

READ PROCESSING:

PRINSEQ—[www.prinseq.sourceforge.net](http://www.prinseq.sourceforge.net)

CLC-genomics workbench

ASSEMBLY:

VELVET—<https://github.com/dzerbino/velvet>

CLC-genomics workbench

SPAdes—<http://bioinf.spbau.ru/spades>

ANNOTATION:

QUAST—<http://bioinf.spbau.ru/quast>

RAST—<http://rast.nmpdr.org>

GenePRIMP—<http://geneprimp.jgi-psf.org>

GLIMMER—<https://ccb.jhu.edu/software/glimmer/>

Prodigal—<http://prodigal.ornl.gov>

QUALITY ASSESSMENT:

QUAST—<http://bioinf.spbau.ru/quast>

CheckM—<https://github.com/Ecogenomics/CheckM>

ProDeGe—https://prodege.jgi-psf.org/

WHOLE GENOME COMPARISON:

ANI—average nucleotide identity—<http://enve-omics.ce.gatech.edu/ani/>

AAI—average amino acid identity—<http://lycofs01.lycoming.edu/~newman/AAI/> and http://enve-omics.ce.gatech.edu/aai/

Mauve—<http://asap.genetics.wisc.edu/software/mauve/>

CAR—contig assembly using rearrangements—<http://genome.cs.nthu.edu.tw/CAR/>

CONTIGuator—<http://contiguator.sourceforge.net>

DDH—digital DNA-DNA hybridization—<http://ggdc.dsmz.de>

**SUPPLEMENTARY TABLES.**

**Table A.** Analysis of raw read files from PacBio and Illumina metagenome sequencing using PRINSEQ.

| **PacBio** | **# sequences** | **Total Bases** | **Mean sequence length** | **Mean GC content** |
| --- | --- | --- | --- | --- |
| **Cathode** |  |  |  |  |
| BW4G1 | 157,574 | 248,518,179 | 1577.15 ± 1004.90 bp | 49.73 ± 8.78 % |
| BW4G2 | 163,725 | 260,050,389 | 1588.34 ± 1011.14 bp | 50.09 ± 8.78 % |
| BW4G3 | 137,567 | 212,303,815 | 1543.28 ± 992.82 bp | 49.59 ± 9.03 % |
| Total | **458,866** | **720,872,383** |  |  |
| **Supernatant** |  |  |  |  |
| BW4S1 | 139,833 | 243,328,679 | 1740.14 ± 1143.87 bp | 48.74 ± 8.73 % |
| BW4S2 | 135,276 | 236,163,107 | 1745.79 ± 1154.44 bp | 49.01 ± 8.79 % |
| BW4S3 | 132,634 | 229,182,208 | 1727.93 ± 1137.73 bp | 48.74 ± 8.72 % |
|  | **407,743** | **708,673,994** |  |  |
|  |  |  |  |  |
| **Illumina MiSeq** | **# sequences** | **Total Bases** | **Mean sequence length** | **Mean GC content** |
| **Cathode** |  |  |  |  |
| BW4G1.R1 | 4,896,577 | 1,161,915,393 | 237.29 ± 36.65 bp | 50.86 ± 10.25 % |
| BW4G1.R2 | 4,896,577 | 1,163,145,718 | 237.54 ± 36.55 bp | 51.00 ± 10.73 % |
| BW4G2.R1 | 5,033,316 | 1,193,544,142 | 237.13 ± 36.92 bp | 50.68 ± 10.21 % |
| BW4G2.R2 | 5,033,316 | 1,194,237,634 | 237.27 ± 36.88 bp | 50.87 ± 10.52 % |
|  | **19,859,786** | **4,712,842,887** |  |  |
| **Supernatant** |  |  |  |  |
| BW4S1.R1 | 2,524,816 | 598,943,299 | 237.22 ± 44.70 bp | 47.17 ± 13.17 % |
| BW4S1.R2 | 2,524,816 | 599,119,844 | 237.29 ± 44.75 bp | 47.30 ± 13.59 % |
| BW4S2.R1 | 2,850,314 | 676,947,919 | 237.50 ± 44.39 bp | 46.97 ± 13.06 % |
| BW4S2.R2 | 2,850,314 | 676,947,919 | 237.50 ± 44.39 bp | 46.97 ± 13.06 % |
|  | **10,750,260** | **2,551,958,981** |  |  |

**Table B.** Assembly statistics from various genome assembly attempts for *Sulfurospirillum sp.* strain MES. Predicted genes were determined using QUAST (A). Multiple gene prediction tools were used for the final draft genome assembly (B).

| Velvet/CLC/SPAdes | **Predicted genes** | **Platform** | **Reference** |
| --- | --- | --- | --- |
|  | 2,655 | GenePRIMP | [2] |
|  | 2,656 | QUAST | [3] |
|  | 2,682 | GeneMarkS+ | [4] |
|  | 2,691 | RAST | [5] |
|  | 2,724 | GLIMMER | [6] |
|  | 2,712 | Prodigal | [7] |
|  | 2,680 | NCBI PGAP | [8] |

| **Assembler(s) used** | **Total number of bases (Mb)** | **GC%** | **# contigs** | **Largest contig** | **N50** | **N75** | **N’s per 100 kbp** | **Predicted genes** |
| --- | --- | --- | --- | --- | --- | --- | --- | --- |
| Velvet/Metavelvet | 2.62 | 43.9 | 51 | 425,125 | 128,597 | 54,519 | 147.57 | 2,659 |
| Velvet/Metavelvet | 2.64 | 43.8 | 58 | 712,535 | 137,638 | 75,977 | 350.96 | 2,711 |
| Velvet/CLC | 2.76 | 43.9 | 67 | 724,237 | 157,982 | 75,922 | 0 | 2,620 |
| Velvet/CLC/SPAdes | 2.67 | 43.8 | 130 | 724,139 | 371,847 | 95,316 | 27.45 | 2,655 |

**Table C.** Genome comparison within the family *Campylobacteraceae.* Data was compiled from the NCBI database [1].

| **Phylogenetic Genus** | **Genome** | **Total bases (Mb)** | **GC%** | **Protein** | **rRNA** | **tRNA** | **Other RNA** | **Gene** | **Pseudogene** |
| --- | --- | --- | --- | --- | --- | --- | --- | --- | --- |
| *Arcobacter* | *Arcobacter butzleri RM4018* | 2.34 | 27.0 | 2,259 | 15 | 54 | -- | 2,333 | 5 |
|  | *Arcobacter nitrofigilis DSM 7299* | 3.19 | 28.4 | 3,126 | 12 | 56 | 2 | 3,220 | 24 |
|  | *Arcobacter cibarius* (assembly) | 2.2 | 27.1 | -- | -- | -- | -- | -- | -- |
|  | *Arcobacter* sp*. L* (gapless chromosome) | 2.95 | 26.6 | 2,845 | 15 | 56 | -- | 2,916 | -- |
|  |  |  |  |  |  |  |  |  |  |
| *Campylobacter* | *Campylobacter coli 1098* | 1.74 | 31.3 | 1,858 | 1 | 30 | -- | 1,889 | -- |
|  | *Campylobacter concisus 13826* | 2.05 | 39.4 | 1,933 | 9 | 46 | 4 | 2,138 | 146 |
|  | *Campylobacter curvus 525.92* | 1.97 | 44.5 | 1,934 | 9 | 46 | 3 | 2,110 | 118 |
|  | *Campylobacter fetus subsp. Fetus 82-40* | 1.77 | 33.3 | 1,719 | 9 | 41 | -- | 1,820 | 50 |
|  | *Campylobacter gracilis RM3268* | 2.26 | 46.6 | 2,847 | 2 | 39 | 3 | 2,891 | 1 |
|  | *Campylobacter hominis ATTC BAA-381* | 1.71 | 31.7 | 1,682 | 9 | 44 | 3 | 1,799 | 61 |
|  | *Campylobacter jejuni subsp. jejuni NCTC* | 1.64 | 30.5 | 1,576 | 9 | 43 | 4 | 1,670 | 38 |
|  | *Campylobacter lari RM2100* | 1.53 | 29.7 | 1,502 | 9 | 46 | 2 | 1,570 | 11 |
|  | *Campylobacter rectus RM3267* | 2.51 | 44.8 | 2,971 | 1 | 39 | 3 | 3,305 | 291 |
|  | *Campylobacter showae RM3277* | 2.07 | 45.7 | 2,361 | 3 | 38 | 3 | 2,405 | -- |
|  | *Campylobacter cuniculorum DSM 23161* (assembly) | 1.87 | 31.2 | -- | -- | -- | -- | -- | -- |
|  | *Campylobacter upsaliensis RM3195* (assembly) | 1.77 | 34.2 | 1,934 | 5 | 43 | 5 | 1,998 | 6 |
|  | *Campylobacter ureolyticus DSM 20703* (assembly) | 1.74 | 28.9 | 1,787 | -- | -- | -- | 1,836 | -- |
|  |  |  |  |  |  |  |  |  |  |
| *Sulfurospirillum* | *Sulfurospirillum barnesii SES-3* | 2.51 | 38.8 | 2,491 | 6 | 41 | 2 | 2,565 | 25 |
|  | *Sulfurospirillum deleyianum DSM 6946* | 2.31 | 39.0 | 2,265 | 9 | 43 | 3 | 2,346 | 26 |
|  | *Sulfurospirillum multivorans DSM 12446* | 3.18 | 40.9 | 3,233 | 6 | 45 | 1 | 3,301 | -- |
|  | *Sulfurospirillum arcachonense* | 2.66 | 30.4 | 2,570 | 5 | 33 | 1 | 2,645 | 36 |
|  | ***Sulfurospirillum cavolei* MES** | **2.67** | **43.8** | **2,485** | **2** | **35** | **1** | **2,656** | **159** |
|  | *Sulfurospirillum* strain Am-N |  |  |  |  |  |  |  |  |
|  | *Sulfurospirillum* sp. SCADC | 2.66 | 41.6 | 2,496 | 2 | 39 | 1 | 2,566 | 28 |
|  | *Sulfurospirillum* strain UCH001 | 2.60 | 37.6 |  | 4 |  |  |  |  |
|  | *Sulfurospirillum cavolei* NBRC | 2.83 | 43.8 | 2,790 | 6 | 46 | 1 | 2,865 | 22 |
|  | *Sulfurospirillum cavolei* UCH003 | 2.698 | 43.9 |  | 3 |  |  |  |  |
|  | *Sulfurospirillum arsenophilum* NBRC | 2.63 | 39.2 | 2,576 | 3 | 39 | 1 | 2,624 | 5 |

**Table D.** Average nucleotide identity (A) and average amino acid identity (B) between *S. cavolei* MES and 10 *Sulfurospirillum* complete and draft genomes.

**A**

| **ANI** | ***S. cav.* MES** | ***S. arc.*** | ***S. bar.*** | ***S. del.*** | ***S. mult.*** | ***S.* sp. SCADC** | ***S.* sp. Am-N** | ***S.* ars. NBRC** | ***S. cav.* NBRC** | ***S. cav.* UCH003** |
| --- | --- | --- | --- | --- | --- | --- | --- | --- | --- | --- |
| ***S. cavolei* MES** | -- | 77.36% | 79.94% | 80.69% | 81.59% | 81.00% | 77.36% | 80.61% | **96.74%** | **96.09%** |
| ***S. arcachonense*** | 77.36% | -- | 79.65% | 79.43% | 79.75% | 79.29% | 80.45% | 79.22% | 81.57% | 77.50% |
| ***S. barnesii*** | 79.94% | 79.65% | -- | 84.33% | 82.02% | 82.35% | 78.04% | 81.84% | 80.28% | 77.62% |
| ***S. deleyianum*** | 80.69% | 79.43% | 84.33% | -- | 81.72% | 81.25% | 77.36% | 81.51% | 80.93% | 78.02% |
| ***S. multivorans*** | 81.59% | 79.75% | 82.02% | 81.72% | -- | 90.06% | 77.02% | 85.19% | 82.17% | 78.66% |
| ***S*. sp. SCADC** | 81.00% | 79.29% | 82.35% | 81.25% | 90.06% | -- | 77.26% | 84.80% | 81.12% | 77.89% |
| ***S.* sp. Am-N** | 77.36% | 80.45% | 78.04% | 77.36% | 77.02% | 77.26% | -- | 77.19% | -- | -- |
| ***S. arsenophilum* NBRC** | 80.61% | 79.22% | 81.84% | 81.51% | 85.19% | 84.80% | 77.91% | -- | 80.71% | 77.73% |
| ***S. cavolei* NBRC** | **96.74%** | 81.57% | 80.28% | 80.93% | 82.17% | 81.12% | -- | 80.71% | -- | 95.18% |
| ***S. cavolei* UCH003** | **96.09%** | 77.50% | 77.62% | 78.02% | 78.66% | 77.89% | -- | 77.73% | 95.18%  95.3%* | -- |
| ***S.* sp*.* UCH001** | 76.97% | 75.79%  69%* | 78.69% | 78.91% | 80.29% | 80.15% | 73.70% | 81.70%  81.4%* | 77.25% | 77.57% |

*From Miura et al., 2015

**B**

| **AAI** | ***S.* MES** | ***S. arc.*** | ***S. bar.*** | ***S. del.*** | ***S. mult.*** | ***S.* sp. SCADC** | ***S.* sp. Am-N** | ***S. ars.* NBRC** | ***S. cav.* NBRC** | ***S. cav.* UCH003** |
| --- | --- | --- | --- | --- | --- | --- | --- | --- | --- | --- |
| ***S. sp.* strain MES** | -- | 61.60 | 75.52 | 76.11 | 77.97 | 78.05 | 62.03 | 77.88 | **97.02** | **96.95** |
| ***S. arcachonense*** | 61.49 | -- | 62.37 | 62.46 | 62.79 | 63.16 | 73.06 | 63.22 | 62.46 | 62.15 |
| ***S. barnesii*** | 75.44 | 62.28 | -- | 84.14 | 78.37 | 78.72 | 61.73 | 78.64 | 78.64 | 75.80 |
| ***S. deleyianum*** | 76.07 | 62.54 | 84.20 | -- | 77.82 | 78.48 | 62.41 | 77.98 | 75.86 | 76.23 |
| ***S. multivorans*** | 77.94 | 62.85 | 78.27 | 77.68 | -- | 92.29 | 62.42 | 86.38 | 78.19 | 78.13 |
| ***S.* sp. SCADC** | 77.94 | 63.27 | 78.88 | 78.57 | 92.28 | -- | 63.19 | 86.38 | 78.44 | 78.18 |
| ***S.* sp*.* Am-N** | 62.12 | 73.16 | 61.76 | 62.71 | 62.35 | 63.04 | -- | 63.53 | 62.44 | 62.56 |
| ***S. arsenophilum* NBRC** | 77.86 | 63.23 | 78.52 | 77.90 | 86.39 | 86.24 | 63.59 | -- | 77.61 | 77.52 |
| ***S. cavolei* NBRC** | **97.01** | 62.47 | 75.88 | 75.74 | 78.21 | 78.43 | 62.49 | 77.47 | -- | 96.12 |
| ***S. cavolei* UCH003** | **96.95** | 62.15 | 75.80 | 76.23 | 78.13 | 78.18 | 62.56 | 77.52 | 96.12 | -- |
| ***S.* sp*.* UCH001** | 78.43 | 63.31 | 78.77 | 78.77 | 85.02 | 85.20 | 63.69 | 87.28 | 77.96 | 78.08 |

**SUPPLEMENTARY FIGURES.**

A)

B)

C)

Cumulative length (kbp)

Number of contigs

# windows

GC%

Contig length (kbp)

% genome coverage

**Figure A.** QUAST quality assessment of SPAdes genome assembly**.** (A) Cumulative length of contigs, (B) GC content, and (C) percent genome coverage.

**A) B)**


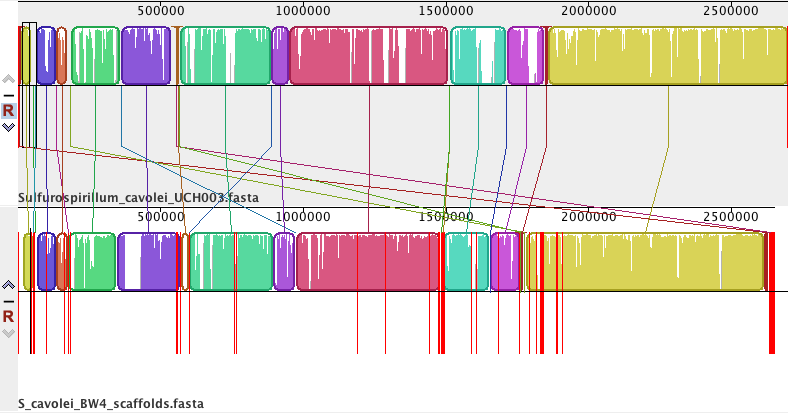
**
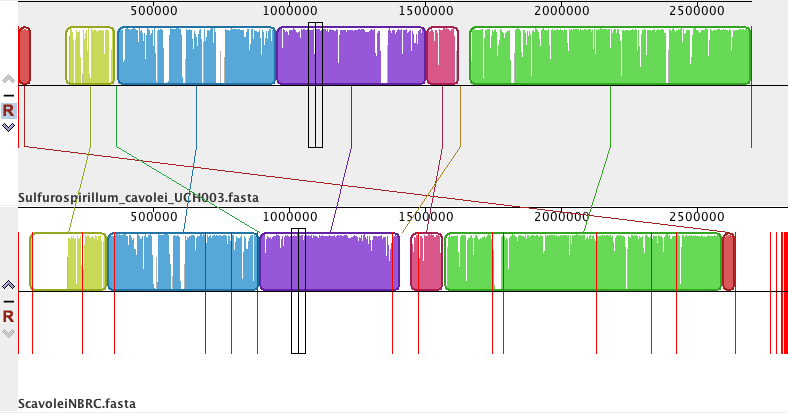
**

**C)**

**
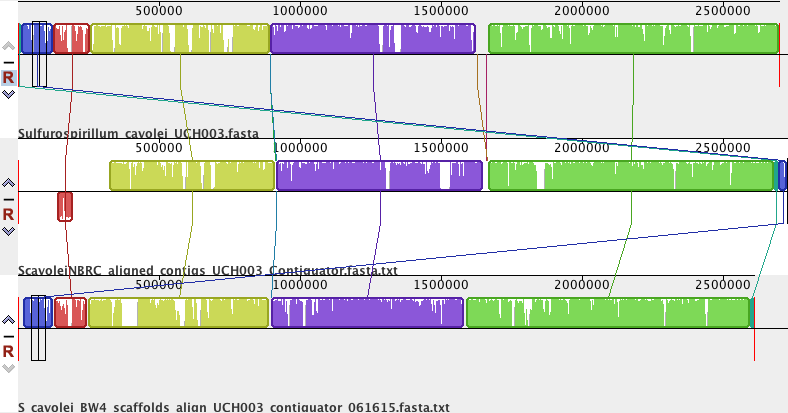
**

**Figure B.** Whole genome alignment of *S. cavolei* UCH003 with draft genome of A) *S. cavolei* MES or B) *S. cavolei* NBRC. Alignments were completed with (top) progressive Mauve [9] and (bottom) Contiguator [10]. C) Whole genome alignment of *S. cavolei* UCH003 (top) with *S. cavolei* NBRC (middle) and *S. cavolei* MES (bottom). Contigs for NBRC or MES were aligned to UCH003 (A and B), concatenated, and aligned with progressive Mauve. See materials and methods for details on alignment parameters.

*S. cavolei* UCH003 (top); *S. cavolei* NBRC (bottom)

*S. cavolei* UCH003 (top); *S. cavolei* MES (bottom)

*S. cavolei* UCH003

*S. cavolei* NBRC

*S. cavolei* UCH003

*S. cavolei* MES

*S. cavolei* NBRC

*S. cavolei* UCH003

*S. cavolei* MES

A B


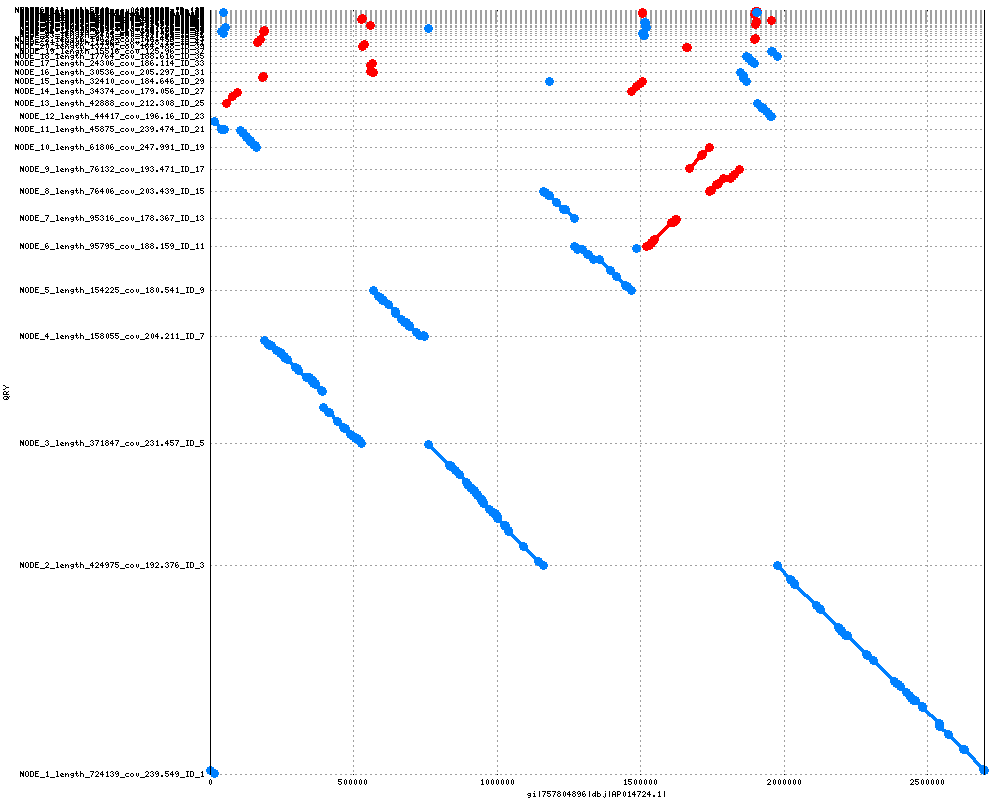

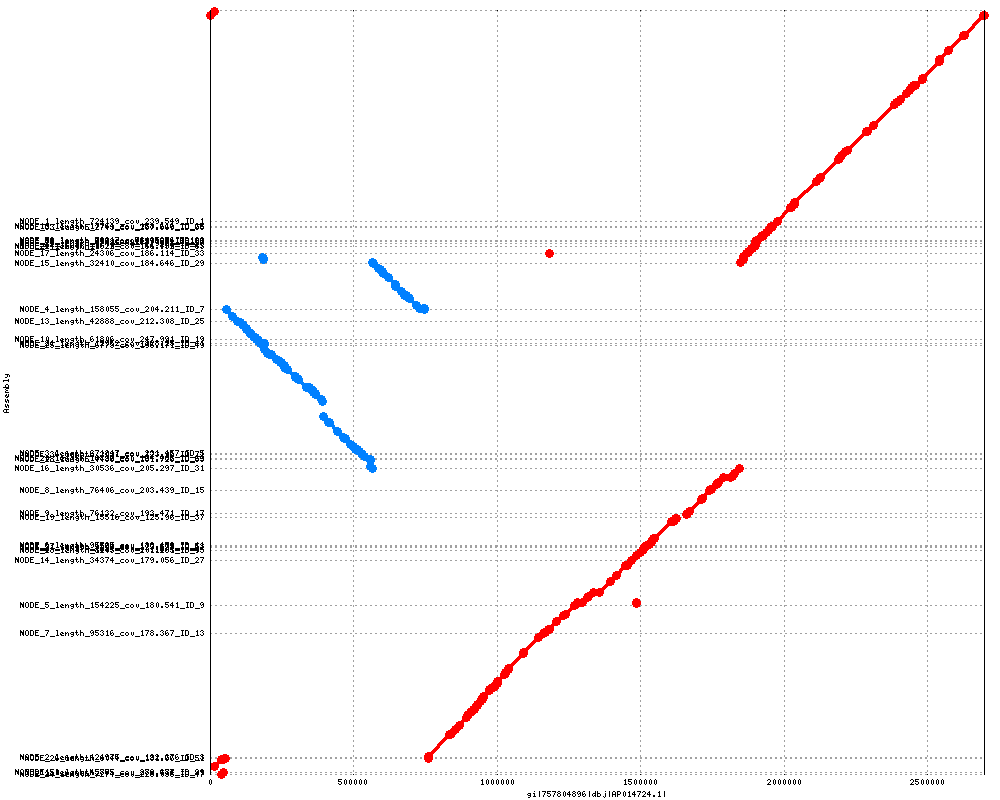


C D


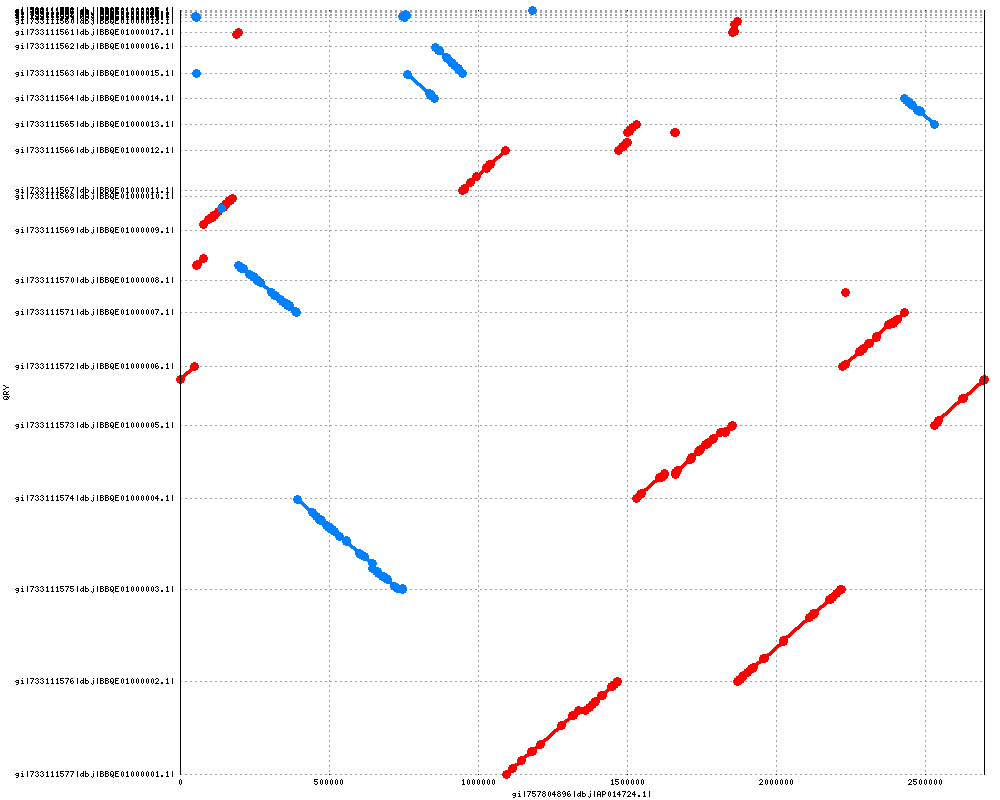

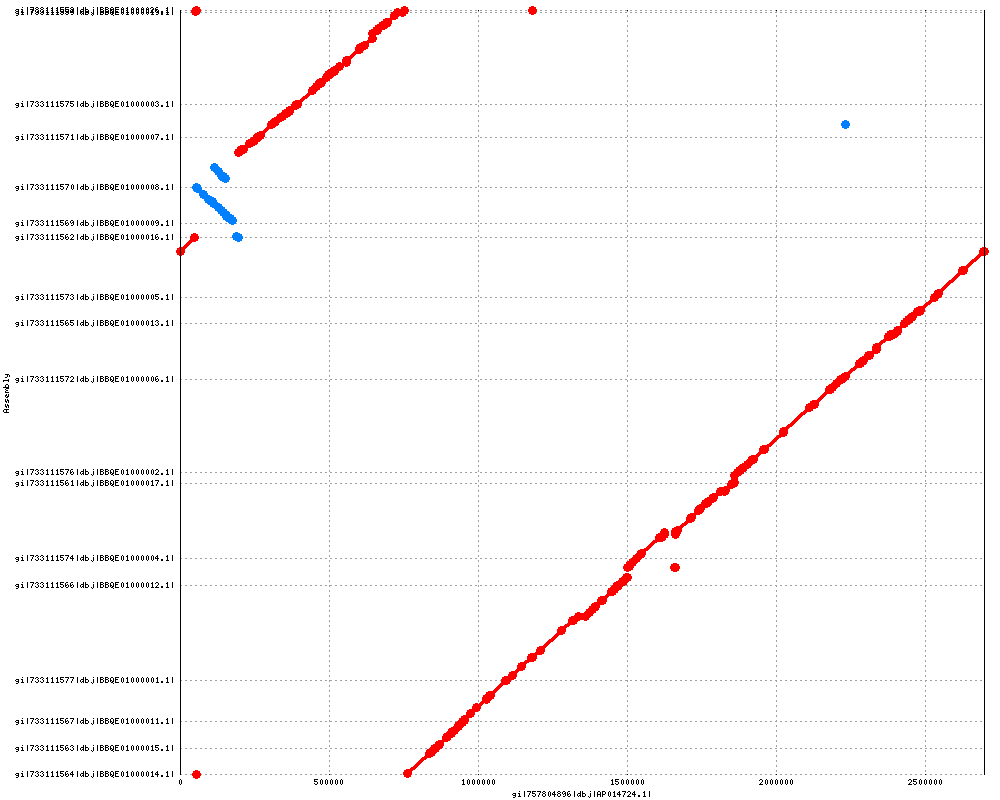


**Figure C.** Dot plot before (A, C) or after (B, D) contig re-arrangement between *S. cavolei* UCH003 and *S. cavolei* MES (A, B) or *S. cavolei* NBRC (C, D).

A) B)


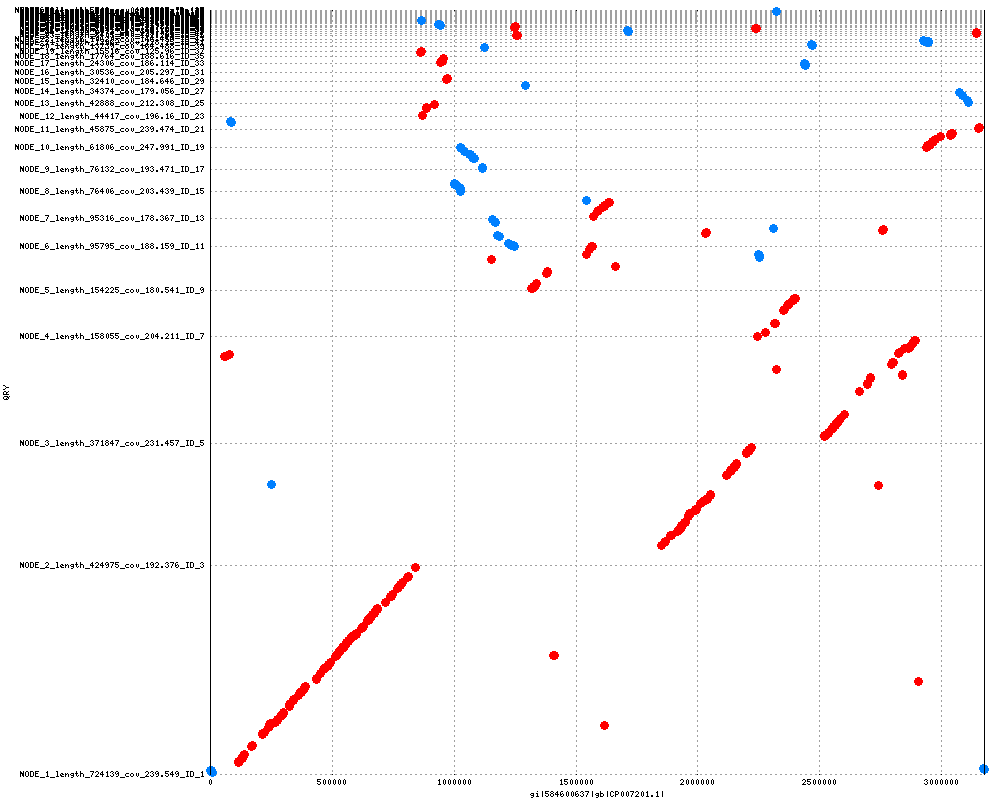
 **
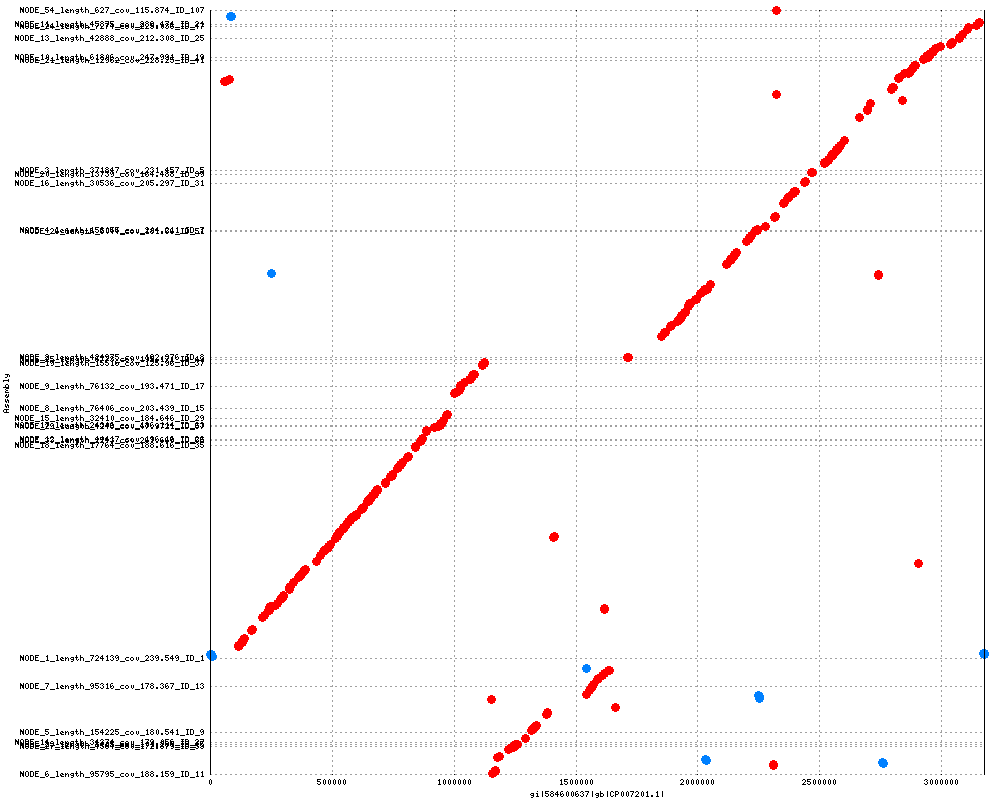
**

C) D)


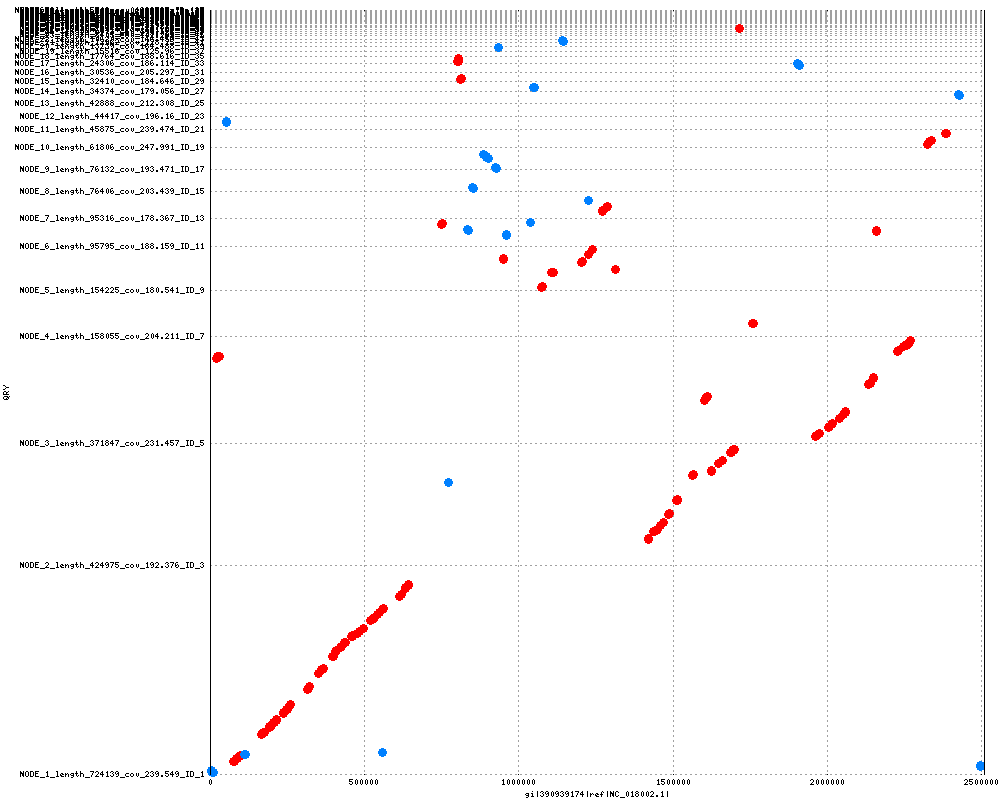

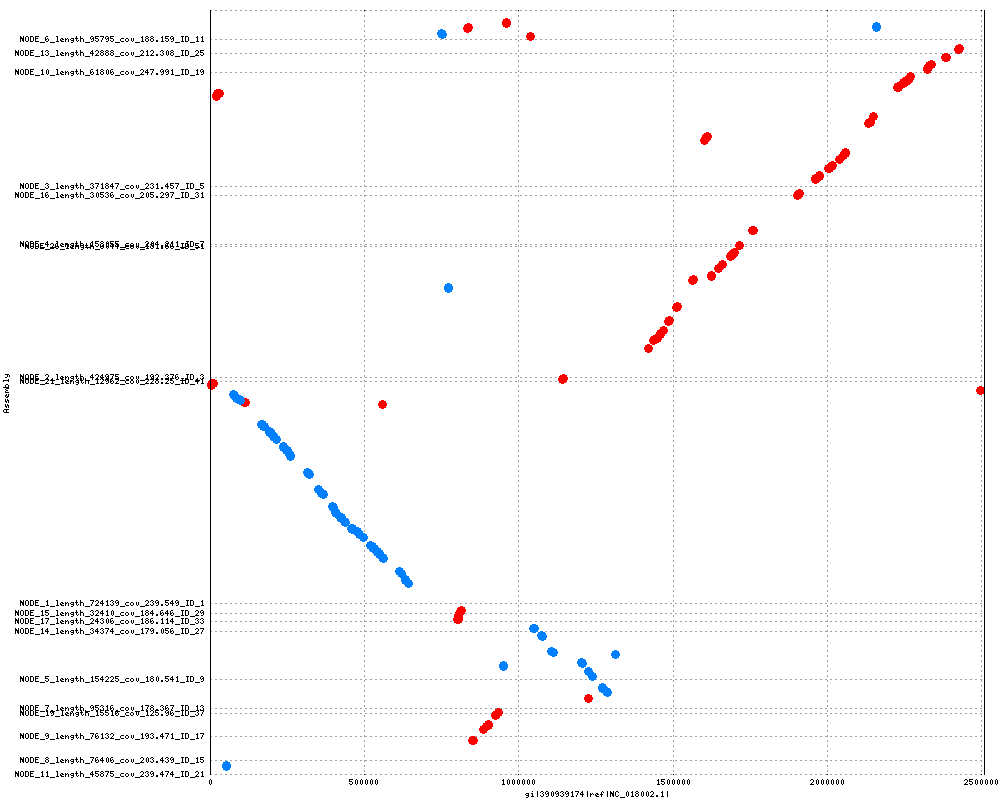


E) F)


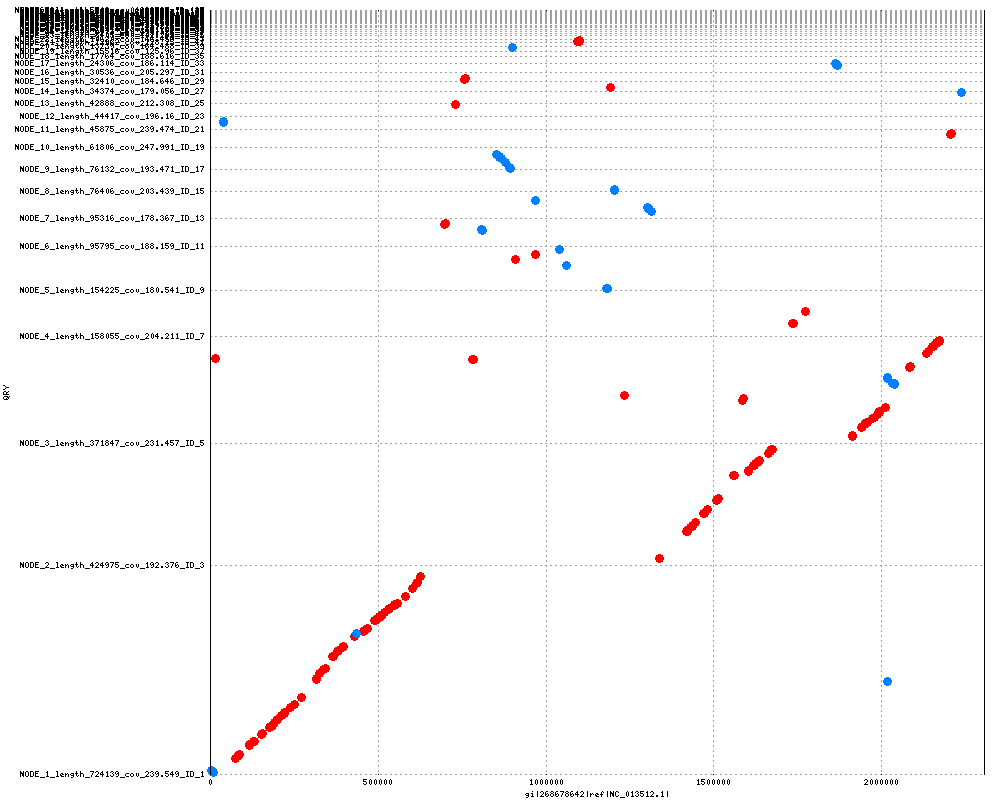

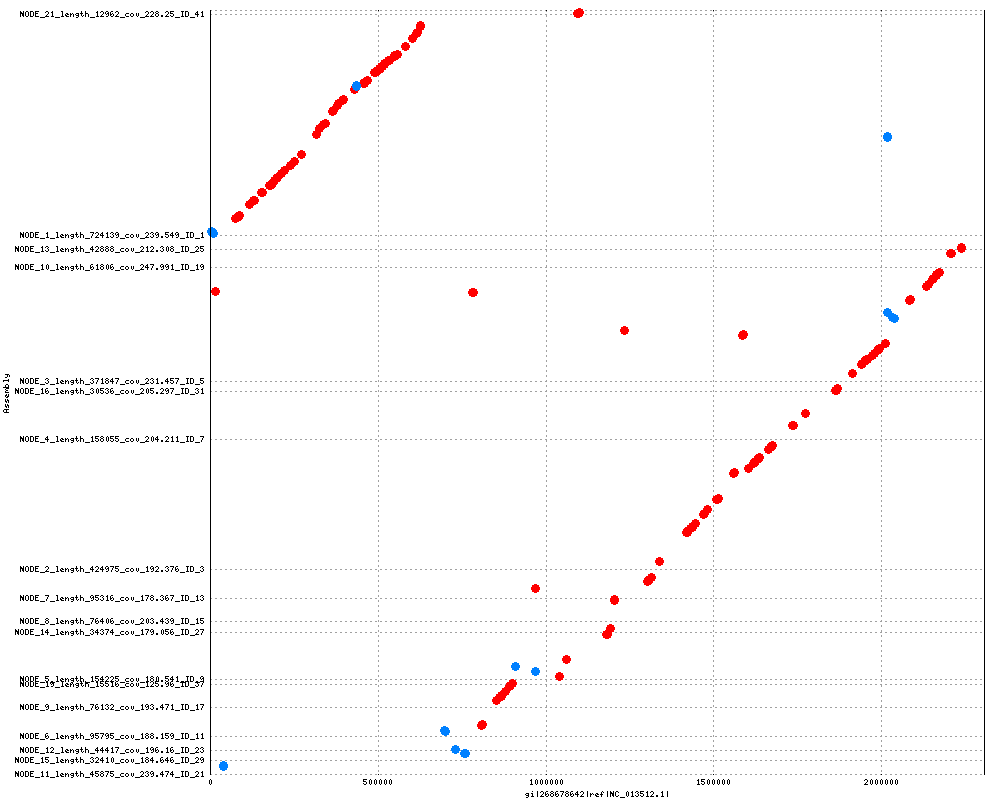


**Figure D.** Whole genome alignment between *S. cavolei* MES (draft) and (A and B) *S. multivorans* (reference), (B and C) *S. barnesii* (reference), and (E and F) *S. deleyianum* (reference) before (A, C, and E) and after (B, D, and F) contig re-arrangement with CAR. Red dots correspond to forward matches while blue dots represent reverse matches.

**Figure E.** Heatmap of Subsystems categories for ten *Sulfurospirillum* proteomes. *Campylobacter curvus* was used for comparison to a non-*Sulfurospirillum* *epsilon proteobacterium* and *E. coli* ATCC 8739 was used for comparison to a non-*epsilon proteobacterium*. Subsystem counts were normalized to total counts per genome. The scale from blue to red represents the Subsystems counts within each category as a percentage of the entire Subsystems counts per genome ranging from 0 to 15%.

A)

B)

**Figure F.** (A) Core-genome and B) pan genome size estimations as a function of the number of genomes (from 1 to 11).


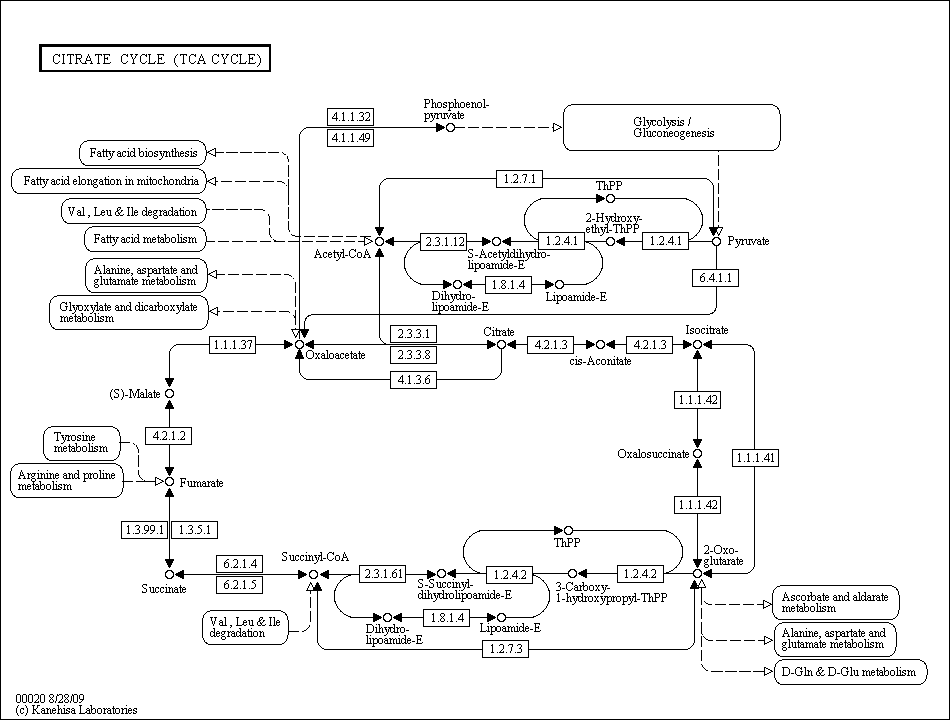


**Figure G.** KEGG pathway for the TCA cycle in *S. cavolei* MES.


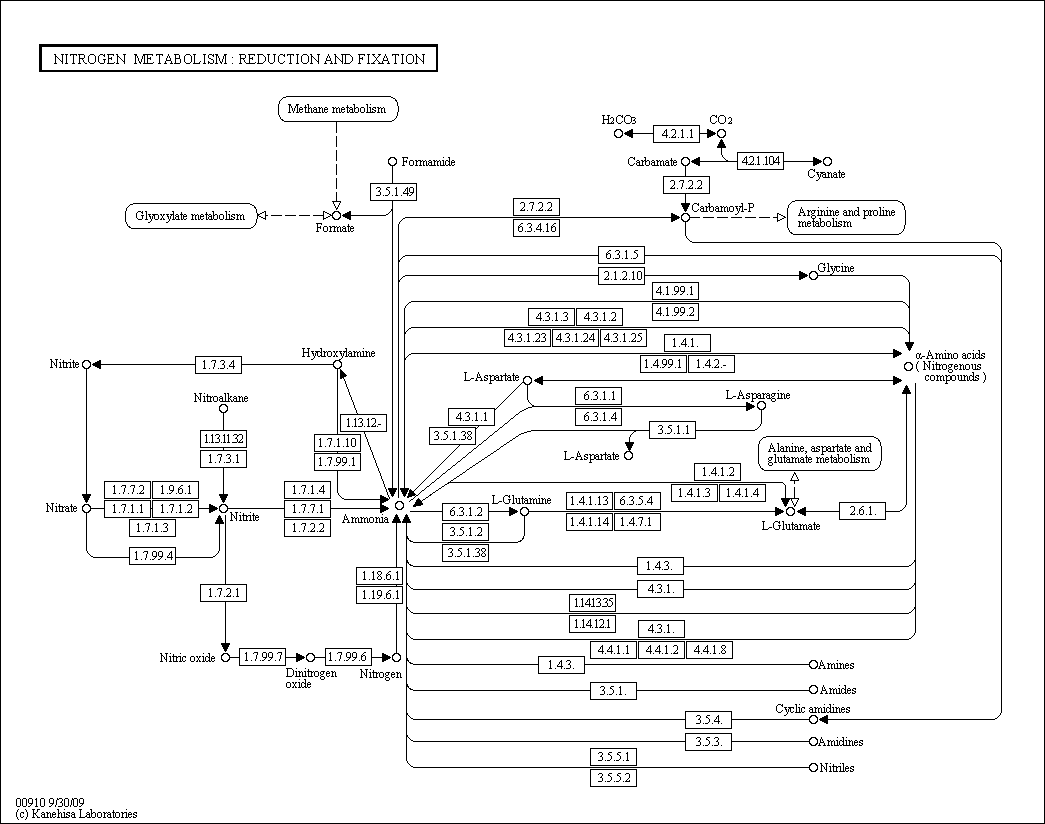


**Figure H.** KEGG pathway for nitrogen metabolism in *S. cavolei* MES.

Sir

AHJ14319.1

KHG33214.1

peg.114|28|53|0.004

peg.276|27|45|1e-38

peg.645|93|96|0.0

AFL69661.1

peg.2092|29|50|0.011

peg.955|90|95|0.0

peg.759|90|95|0.0

peg.133|90|95|0.0

KFL33139.1

peg.2455|90|95|0.0


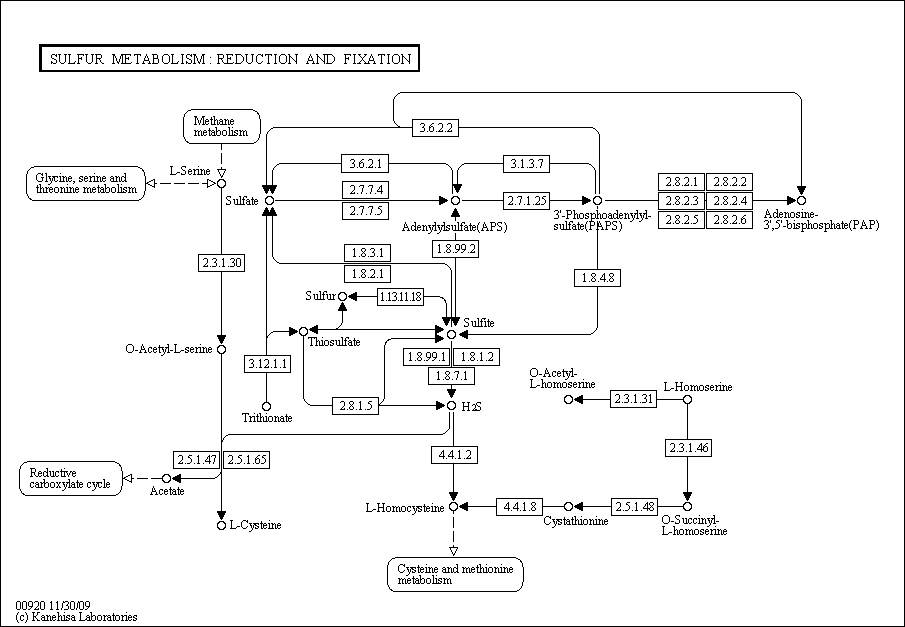


Assimilatory sulfate reduction

Sulfate APS PAPS Sulfite Sulfide

**Figure I.** KEGG pathway for sulfur metabolism in *S. cavolei* MES. The assimilatory sulfate reduction pathway is shown in detail with available Genbank protein IDs. The RAST idendifier and sequence identity, positives, and e-value [based upon BLASTP results against Sat, CysN, CysD, CysH or Sir from *S. multivorans* or MccA (UniProt:Q7MSJ8) from *Wolinella succinogens*] are shown when Genbank IDs were unavailable.

*S. multivorans*

*S. cavolei* MES

*S.* sp. Am-N

*S. arcachonense*

*S. arsenophilum*

*S. barnesii*

*S. deleyianum*

*S. cavolei* NBRC

*S. cavolei* UCH003

*S.* sp. SCADC

*S.* sp. UCH001

MccA

AHJ14079.1

KHG33545.1

peg.1726|27|41|0.004

peg.1266|62|77|0.0

peg.856|85|91|0.0

AFL68074.1

ACZ11738.1

peg.1633|86|93|0.0

peg.1581|86|93|0.0

KFL34660.1

peg.2280|85|91|0.0

CysH

AHJ14313.1

KHG33209.1

peg.1448|46|60|0.38

peg.273|42|63|1e-59

peg.651|92|97|1e-163

AFL69655.1

peg.1265|23|45|0.20

peg.961|87|93|e-153

peg.765|87|93|e-153

peg.139|85|92|e-150

KFL33145.1

peg.2449|87|93|e-153

CysD

AHJ14315.1

peg.231|38|52|8e-63

peg.274|36|53|3e-63

peg.650|97|98|0.0

AFL69657.1

peg.1357|35|60|3.5

peg.960|96|98|0.0

peg.764|96|98|0.0

peg.138|96|98|0.0

KFL33143.1

peg.2450|97|98|0.0

CysN

AHJ14314.1

KHG33210.1

peg.229|36|57|3e-83

peg.275|37|58|2e-98

peg.649|97|99|0.0

AFL69656.1

peg.349|26|50|3e-28

peg.959|92|96|0.0

peg.763|92|96|0.0

peg.137|92|97|0.0

KFL33144.1

peg.2451|93|96|0.0

Sat

AHJ13651.1

KHG33739.1

peg.2094|74|85|0.0

peg.1804|70|85|0.0

peg.188|95|98|0.0

AFL69069.1

ACZ12724.1

peg.1858|90|96|0.0

peg.678|90|96|0.0

KFL33819.1

peg.1918|95|98|0.0

CysN

Sat

CysND

CysH

Sir/MccA

CysJI

**EMIRGE full-length 16S rRNA gene of *S. cavolei* MES reconstructed from metagenome sequences**

**Figure J.** Comparison of gene synteny of the periplasmic [Fe] hydrogenase from *S. cavolei* MES, *S. cavolei* NBRC, and *S. cavolei* UCH003. Numbers represent the length of each predicted translated protein (in amino acids).

AGAGTTTGATCCTGGCTCAGAGTGAACGCTGGCGGCGTGCTTAACACATGCAAGTCGAACGGA

TGAAATAAGCTTGCTTATTTCGTTAGTGGCGCACGGGTGAGTAATGTATAGCTAACCTGCCCTTTAGTGGGGGACAACAGATGGAAACGTCTGCTAATACCCCATACTCCTGCGTATCATAAGATACGTTGGGAAAGATTTATTGCTAAAGGATGGGGCTTTATGGTATCAGCTAGTTGGTGGGGTAACGGCCTACCAAGGCAATGACGCCTACCTGGTCTGAGAGGATGATCAGGCACACTGGAACTGAGACACGGTCCAGACTCCTACGGGAGGCAGCAGTGGGGAATATTGCACAATGGGGGAAACCCTGATGCAGCAACGCCGCGTGGAGGATGACGCATTTCGGTGTGTAAACTCCTTTTATAAGGGAAGATAATGACGGTACCTTATGAATAAGCACCGGCTAACTCCGTGCCAGCAGCCGCGGTAATACGGAGGGTGCAAGCGTTACTCGGAATCACTGGGCGTAAAGGATGCGTAGGCTGTAATATAAGTCAGAAGTGAAATCCAACGGCTTAACCGTTGAACTGCTTTTGAAACTGTTTTACTAGAATATGGGAGAGGTAGATGGAATTGGTGGTGTAGGGGTAAAATCCGTAGATATCACCAGGAATACCGATTGCGAAGGCGATCTACTGGAACATTATTGACGCTGAGGCATGAAAGCGTGGGGAGCAAACAGGATTAGATACCCTGGTAGTCCACGCCCTAAACGATGCACACTAGTTGTTGCGATGCTAGTCATTGCAGTAATGCACTTAACAGATTAAGTGTGCCGCCTGGGGAGTACGGTCGCAAGATTAAAACTCAAAGGAATAGACGGGGACCCGCACAAGCGGTGGAGCATGTGGTTTAATTCGAAGATACACGAAGAACCTTACCTGGGCTTGATATCCTTGGAATCTTGTAGAGATACAAGAGTGCTAGTTTACTAGAACCAAGAGACAGGTGCTGCAC

GGCTGTCGTCAGCTCGTGTCGTGAGATGTTGGGTTAAGTCCCGCAACGAGCGCAACCCTCGTGATTAGTTGCTAACAGTTTGGCTGAGCACTCTAATCAGACTGCCTTCGCAAGGAGGAGGAAGGTGAGGACGACGTCAAGTCATCATGGCCCTTATGCCCAGGGCTACACACGTGCTACAATGGCGCGTACAAAGAGAGGCGATACCGCGAGGTGGAGCAAATCTTAAAAACGCGTCTCAGTTCGGATTGGAGTCTGCAACTCGACTCCATGAAGCTGGAATCGCTAGTAATCGTAGATCAGATATGCTACGGTGAATACGTTCCCGGGTCTTGTACTCACCGCCCGTCACACCATGGGAGTTGAATTCACCCGAAGCCGGAATACTAAACTAGTTACCGACCACGGTGGGTTCAGCGACTGGGGTGAAGTCGTAACAAGGTAACCGTAGGAGAACCTGCGGTTGGATCACCTCCT

References

1. Benson DA, Clark K, Karsch-Mizrachi I, Lipman DJ, Ostell J, et al. (2014) GenBank. Nucleic Acids Res 42: D32–D37.

2. Pati A, Ivanova NN, Mikhailova N, Ovchinnikova G, Hooper SD, et al. (2010) GenePRIMP: a gene prediction improvement pipeline for prokaryotic genomes. Nat Methods 7: 455–457.

3. Gurevich A, Saveliev V, Vyahhi N, Tesler G (2013) QUAST: quality assessment tool for genome assemblies. Bioinformatics 29: 1072–1075.

4. Besemer J, Lomsadze A, Borodovsky M (2001) GeneMarkS: a self-training method for prediction of gene starts in microbial genomes. Implications for finding sequence motifs in regulatory regions. Nucleic Acids Res 29: 2607–2618.

5. Aziz RK, Bartels D, Best AA, DeJongh M, Disz T, et al. (2008) The RAST Server: rapid annotations using subsystems technology. BMC Genomics 9: 75.

6. Delcher A (1999) Improved microbial gene identification with GLIMMER. Nucleic Acids Res 27: 4636–4641.

7. Hyatt D, Chen G-L, Locascio PF, Land ML, Larimer FW, et al. (2010) Prodigal: prokaryotic gene recognition and translation initiation site identification. BMC Bioinformatics 11: 119.

8. Angiuoli S V, Gussman A, Klimke W, Cochrane G, Field D, et al. (2008) Toward an online repository of Standard Operating Procedures (SOPs) for (meta)genomic annotation. OMICS 12: 137–141.

9. Rissman AI, Mau B, Biehl BS, Darling AE, Glasner JD, et al. (2009) Reordering contigs of draft genomes using the Mauve Aligner. Bioinformatics 25: 2071–2073.

10. Galardini M, Biondi EG, Bazzicalupo M, Mengoni A (2011) CONTIGuator: a bacterial genomes finishing tool for structural insights on draft genomes. Source Code Biol Med 6: 11.

11. Lu C, Chen K-T, Huang S-Y, Chiu H-T (2014) CAR: contig assembly of prokaryotic draft genomes using rearrangements. BMC Bioinformatics 15: 381.
